# Associations between obstructive sleep apnea and Alzheimer's disease-related biomarkers (amyloid-β and tau) in blood and cerebrospinal fluid: a systematic review and meta-analysis

**DOI:** 10.3389/fneur.2026.1817426

**Published:** 2026-09-21

**Authors:** Jing Wang, Ligeer Che, Rina Su, Xianbin Wang

**Affiliations:** 1 Baotou Medical College, Inner Mongolia University of Science & Technology, Baotou, China; 2 The Second Affiliated Hospital of Baotou Medical College, Inner Mongolia University of Science & Technology, Baotou, China

**Keywords:** Alzheimer's disease, blood biomarkers, cerebrospinal fluid biomarkers, meta-analysis, obstructive sleep apnea, systematic review

## Abstract

**Purpose:**

This study aimed to clarify the relationship between obstructive sleep apnea (OSA) and Alzheimer's disease (AD)-related biomarkers—specifically amyloid β (Aβ) and tau proteins—in blood and cerebrospinal fluid (CSF) through a systematic review and meta-analysis.

**Methods:**

PubMed, Web of Science (WOS), Embase, and Cochrane Library were searched from database inception to December 30, 2024 (PROSPERO registration: CRD420251148709). Studies reporting blood or CSF levels of Aβ40, Aβ42, total Aβ (*T*-Aβ), total tau (*T*-tau), or phosphorylated tau (*P*-tau) in patients with OSA vs. non-OSA controls were included. A random-effects model was employed for all meta-analyses. The risk of bias was assessed using appropriate validated tools.

**Results:**

Thirteen studies (seven blood, six CSF) were included. In blood, OSA patients showed significantly higher *T*-Aβ (SMD = 0.66, 95% CI [0.34, 0.97], *I*^2^ = 0%, *p* < 0.01) and *T*-tau (SMD = 1.48, 95% *C*I [0.33, 2.62], *I*^2^ = 93.6%, *p* = 0.011). No significant differences were found for blood Aβ40, Aβ42, Aβ42/Aβ40 ratio, or *P*-tau. In CSF, patients with OSA had significantly lower Aβ40 (SMD = −1.13, 95% CI [−1.65, −0.60], *I*^2^ = 0%, *p* < 0.01) and Aβ42 (SMD = −1.23, 95% CI [−1.84, −0.63], *I*^2^ = 77.3%, *p* < 0.01), but no differences in Aβ42/Aβ40 ratio, *T*-tau, or *P*-tau.

**Limitations:**

High heterogeneity, pre-dominantly cross-sectional designs, low-to-very-low GRADE certainty for most outcomes, retrospective registration, and limited study numbers preclude causal inference and warrant cautious interpretation.

**Conclusions:**

This exploratory meta-analysis revealed compartment-specific associations between certain AD-related biomarkers and OSA. However, given the low-to-very-low certainty of the available evidence and the substantial uncertainty reflected by prediction intervals that frequently crossed the null value, these findings should be regarded as hypothesis-generating rather than conclusive. Notably, the absence of consistent alterations in *P*-tau or the CSF Aβ42/Aβ40 ratio argues against a direct signature of established AD neuropathology. Future rigorously designed, large-scale prospective studies are warranted to determine whether these biomarker changes reflect early AD pathology or reversible physiological responses to OSA-related hypoxia and sleep fragmentation.

**Systematic review registration:** https://www.crd.york.ac.uk/prospero/display_record.php?RecordID=1148709, identifier CRD420251148709.

## 1 Introduction

Obstructive sleep apnea (OSA) is a common sleep-related breathing disorder characterized by recurrent upper airway collapse during sleep, leading to intermittent hypoxia and sleep fragmentation (1, 2). Intermittent hypoxia and repeated arousals have been shown to be closely associated with memory impairment and neurocognitive deficits in patients with OSA (3, 4). The link between OSA and cognitive dysfunction has been extensively investigated, with oxidative stress and inflammatory responses induced by intermittent hypoxia recognized as key mechanisms underlying cognitive decline (5). In addition, the association between OSA and neurodegenerative diseases, such as Alzheimer's disease (AD), has attracted increasing attention. Evidence suggests that the prevalence of OSA is markedly elevated among patients with AD, indicating that OSA may represent a potentially modifiable risk factor for Alzheimer's disease and related dementias (6).

Abnormal deposition of amyloid-β (Aβ) and tau protein in the brain constitutes the hallmark pathological features of neurodegeneration (7). The levels of Aβ and tau protein in blood and CSF have been widely recognized as important biomarkers of AD and other neurodegenerative diseases (8). Emerging studies have preliminarily revealed alterations in Aβ and tau protein levels in both CSF and blood of patients with OSA. For example, some investigations have reported decreased CSF Aβ42 levels in OSA patients compared with controls, whereas others have identified elevated plasma Aβ42 levels (9, 10). However, the findings remain inconsistent, with some studies reporting no significant association between OSA and changes in blood or CSF biomarkers (11).

Therefore, to address these inconsistencies, we formulated a structured research question following the PICO framework: among adult individuals (Population), does the presence of obstructive sleep apnea (Exposure), compared to non-OSA controls (Comparator), show an association with the levels of Aβ and tau proteins in blood or cerebrospinal fluid (Outcomes)? Accordingly, the present study aimed to evaluate, through a comprehensive systematic review and meta-analysis, the association between OSA and the levels of neurodegenerative biomarkers in plasma and CSF, thereby providing a quantitative synthesis of the available evidence.

## 2 Materials and methods

In conducting this systematic review and meta-analysis, we adhered to the Preferred Reporting Items for Systematic Reviews and Meta-Analyses (PRISMA) recommendations (12). We ensured prospective registration in the PROSPERO database (CRD420251148709). The review protocol is available from the corresponding author upon reasonable request. No amendments were made to the protocol after registration. We acknowledge that registration was completed retrospectively (September 18, 2025) after the literature search (December 30, 2024) had been performed. The study protocol was developed prior to data extraction and statistical analysis, and the primary research question, search strategy, inclusion/exclusion criteria, and outcome definitions remained unchanged from the protocol. No deviations from the pre-specified analysis plan occurred. Full transparency regarding the retrospective nature of registration is provided here to comply with methodological best practices.

### 2.1 Eligibility criteria

#### 2.1.1 Participants

OSA group: patients diagnosed with OSA defined by an Apnea-Hypopnea Index (AHI) ≥5 events/hour, in accordance with the clinical practice guidelines established by the American Academy of Sleep Medicine (AASM) (13), with diagnosis confirmed by overnight in-laboratory polysomnography (PSG) or home sleep apnea testing (HSAT). Studies relying solely on clinical symptoms, screening questionnaires, or limited-channel devices without AHI quantification were excluded. Control group: individuals with AHI < 5 events/hour, either non-OSA patients or healthy participants.

#### 2.1.2 Cognitive status of participants

We extracted information on participants' cognitive status from each included study. Among the 13 included studies:

Nine studies explicitly reported that participants were cognitively normal (based on Mini-Mental State Examination [MMSE] score ≥26 or Montreal Cognitive Assessment [MoCA] score ≥26, or clinical evaluation). Two studies included participants with mild cognitive impairment (MCI), but reported biomarker data separately or adjusted for cognitive status. Two studies did not report any cognitive assessment. No studies included participants with established dementia (e.g., Alzheimer's disease, vascular dementia). The cognitive screening tools used across studies included MMSE (*n* = 7), MoCA (*n* = 3), or a clinical interview without a formal scale (*n* = 1).

#### 2.1.3 Study design

We included cross-sectional and cohort studies that reported neurodegenerative biomarker levels in blood or CSF among patients with OSA compared with healthy controls.

#### 2.1.4 Outcomes

##### 2.1.4.1 Primary outcomes

Blood: total amyloid-β (*T*-Aβ) and total tau (*T*-tau)

CSF: amyloid-β42 (Aβ42) and amyloid-β40 (Aβ40).

##### 2.1.4.2 Secondary outcomes

Blood: amyloid-β40 (Aβ40), amyloid-β42 (Aβ42), Aβ42/Aβ40 ratio, and phosphorylated tau (*P*-tau);

CSF: Aβ42/Aβ40 ratio, total tau (*T*-tau), and phosphorylated tau (*P*-tau).

##### 2.1.4.3 Rationale for outcome prioritization

Primary outcomes were selected based on their established roles as core AD pathological hallmarks and their frequent reporting in the included studies. *T*-Aβ and *T*-tau in blood were prioritized due to their non-invasive sampling potential, while CSF Aβ42 and Aβ40 were prioritized given their validated diagnostic utility for cerebral amyloid deposition (4).

##### 2.1.4.4 Exclusion criteria

(1) Duplicate publications; (2) reviews, case reports, *in vitro* studies, pediatric studies, or narrative/systematic reviews; (3) studies with inaccessible full texts, incomplete clinical data, or erroneous data that could not be clarified by contacting the authors; (4) studies using self-defined diagnostic criteria or outcome measures; (5) conference abstracts; (6) non-English publications.

### 2.2 Literature search strategy

We systematically searched PubMed, Web of Science, Embase, and the Cochrane Library for studies reporting neurodegenerative biomarkers in blood or CSF of patients with OSA, from database inception to December 30, 2024. The main search terms included: (“Sleep Apnea, Obstructive” OR “Obstructive Sleep Apnea”) AND (“Amyloid beta-Peptides” OR “tau Proteins”). The complete search strategy is provided in Supplementary file 1.

To ensure search comprehensiveness, we adopted the following strategies. First, the selected databases represent the core resources for biomedical systematic reviews: PubMed (biomedical literature), Web of Science (multidisciplinary coverage), Embase (extensive European and pharmaceutical literature), and Cochrane Library (controlled trials and systematic reviews). Second, we combined controlled vocabulary (MeSH terms for PubMed; Emtree terms for Embase) with free-text keywords to maximize sensitivity. Third, the search terms were developed iteratively by reviewing key published studies in this field and consulting a medical librarian. Fourth, we did not apply language or publication type restrictions beyond the exclusion criteria. We verified that all pre-specified key studies (e.g., Bu et al., (38); Liguori et al., (9)) were captured by the search.

### 2.3 Study selection and data extraction

All search results were imported into Zotero reference management software, and duplicate records were removed. Two trained investigators independently screened studies in two stages. In the initial screening, titles and abstracts were reviewed to exclude irrelevant studies. In the full-text screening, potentially eligible studies were assessed against inclusion and exclusion criteria, with reasons for exclusion recorded. Data were extracted using a standardized form, including author, year of publication, sample size, mean age, sex distribution, body mass index (BMI), AHI, oxygen desaturation index (ODI), total sleep time (TST), sleep efficiency (SE), mean oxyhaemoglobin saturation (Mean_SaO_2_), and oxyhemoglobin saturation nadir (SaO_2_ nadir). The data extraction form was piloted on two randomly selected studies by two reviewers independently. Minor adjustments were made to the form to improve clarity and consistency before formal data extraction. Disagreements were resolved by consensus or adjudication by a third reviewer. For missing data, we attempted to contact study authors. If unavailable, the study was excluded. Among the excluded studies, one study was excluded because it reported biomarker levels as medians with interquartile ranges (IQRs) rather than means with standard deviations. We attempted to convert the reported medians and IQRs to means and SDs using the validated method by Wan et al. (14) and Luo et al. (15). However, the study did not report the minimum and maximum values required for accurate conversion, and attempts to contact the authors for raw data were unsuccessful. Under these circumstances, we made the conservative decision to exclude this study from the quantitative synthesis to avoid introducing potentially inaccurate converted estimates.

### 2.4 Risk of bias assessment

Two investigators independently assessed risk of bias using the Risk Of Bias In Non-randomized Studies of Exposures (ROBINS-E) tool. This checklist evaluates seven domains: confounding, exposure measurement, selection of participants, post-exposure interventions, missing data, outcome measurement, and selective reporting. Each domain and the overall risk were categorized as low, some concerns, High, or No information (16). To incorporate risk-of-bias assessments into the synthesis and interpretation of results, we performed the following analyses: (1) sensitivity analyses excluding studies rated as high or critical risk of bias to assess the robustness of pooled estimates; (2) subgroup analyses stratified by overall risk of bias (low/moderate vs. serious/critical) when sufficient studies were available (≥2 per subgroup); and (3) visual inspection of funnel plots to assess publication bias, with Egger's regression test applied only when ≥10 studies were included, given its limited statistical power with fewer studies (17, 18). The results of these sensitivity and subgroup analyses are reported alongside the main meta-analyses in the Results section. Publication bias was assessed by visual inspection of funnel plots (19, 20).

### 2.5 Statistical analysis

All analyses were performed using RStudio (Version 4.4.3) with the “meta” and “metafor” packages. Continuous variables were summarized as means and standard deviations (SD). Heterogeneity was quantified using *I*^2^, with values of 25%, 50%, and 75% representing low, moderate, and high heterogeneity, respectively (21).

We used random effects models for all meta-analyses (22). This decision was made *a priori*, regardless of the observed *I*^2^ values, for the following reasons (23). First, we anticipated substantial clinical and methodological heterogeneity across studies due to differences in OSA severity (mild vs. moderate vs. severe), biomarker assay methods (ELISA, SIMOA, etc.), sample types (plasma vs. serum vs. CSF), and population characteristics (age, BMI, comorbidities). Random-effects models account for both within-study and between-study variance, providing more conservative estimates when heterogeneity is present (24). Second, fixed-effect models assume that all studies share a single common true effect size (25), an assumption that is rarely justified in meta-analyses of observational studies (26). Third, when heterogeneity is low (*I*^2^ < 25%), random-effects models yield results nearly identical to fixed-effect models; when heterogeneity is high, random-effects models provide wider confidence intervals that better reflect the uncertainty (24). Therefore, adopting a uniform random-effects model represents a principled and conservative approach that does not risk overconfidence in the pooled estimates (23). The between-study variance (τ^2^) was estimated using the restricted maximum likelihood (REML) method (27), an iterative estimator that accounts for the uncertainty in the fixed-effect coefficients. Compared with the DerSimonian-Laird method, REML generally provides less biased τ^2^ estimates and more accurate standard errors for regression coefficients, particularly when the number of studies is small or heterogeneity is substantial (27). For outcomes with *I*^2^ > 90%, pooled estimates were interpreted as exploratory, with emphasis placed on prediction intervals and sensitivity analyses rather than on the point estimate alone (26). Given the small number of studies per outcome (*k* < 5 for most analyses), the Hartung-Knapp adjustment was not applied as it may yield unstable estimates; however, sensitivity analyses using this adjustment yielded consistent results (data not shown) (28).

Prediction intervals: for outcomes with substantial heterogeneity (*I*^2^ ≥ 50%), we calculated 95% prediction intervals (PIs) to estimate the range within which the true effect size of a future similar study would fall (29, 30). The prediction interval was computed using the formula: PI = pooled effect ± *t* {k−2} × √(τ^2^ + SE {pooled}^2^), where k is the number of studies. PIs complement confidence intervals by providing information about the expected dispersion of effects across different settings, which is particularly important when heterogeneity is present (31).

All meta-analyses were conducted using the inverse variance method. When sufficient data were available, meta-regression was conducted to explore associations between baseline characteristics and biomarker levels. Permutation tests were applied to reduce false-positive results (32). A *p*-value < 0.05 was considered statistically significant.

To explore potential sources of heterogeneity and assess the robustness of the pooled estimates, we conducted sensitivity analyses for outcomes with substantial heterogeneity (*I*^2^ ≥ 75%) or limited numbers of included studies (fewer than 5). Specifically, we performed leave-one-out sensitivity analyses, sequentially removing each study and recalculating the pooled effect size and *I*^2^ to identify whether any single study was disproportionately driving heterogeneity (33). If the leave-one-out analysis identified a specific study as a major source of heterogeneity (i.e., its exclusion substantially reduced the *I*^2^ value), we conducted an additional sensitivity analysis excluding that influential study. We re-ran the random-effects model to compare the pooled estimates with and without that study.

### 2.6 Certainty of evidence assessment (GRADE)

We assessed the overall certainty of evidence for all primary and secondary outcomes that underwent meta-analysis using the Grading of Recommendations Assessment, Development and Evaluation (GRADE) framework (34, 35). The GRADE approach evaluates evidence across five domains that can lower certainty: risk of bias (assessed using ROBINS-E, Section 2.4), inconsistency (evaluated based on *I*^2^ statistic, overlap of confidence intervals, and prediction intervals when calculated), indirectness (assessed regarding population, exposure, comparator, and outcomes), imprecision (judged based on the optimal information size [OIS] and the width of 95% confidence intervals relative to clinically meaningful effect thresholds), and publication bias (assessed using funnel plot asymmetry) (36). Evidence certainty was rated as high (⊕⊕⊕⊕), moderate (⊕⊕⊕⊖), low (⊕⊕⊖⊖), or very low (⊕⊖⊖⊖) according to GRADE guidelines (35). Two investigators independently rated the evidence, with disagreements resolved by consensus. The full GRADE assessments for all meta-analyzed outcomes are summarized in a summary of findings table and incorporated into the interpretation of results (37).

## 3 Results

### 3.1 Literature screening and selection

A total of 382 records were retrieved based on the pre-defined search strategy. After removing duplicates and screening titles and abstracts, 368 studies were excluded. The remaining studies were assessed in full text according to the eligibility criteria, resulting in 14 studies initially considered for inclusion. However, the study by Sharma et al. was excluded because it reported medians with interquartile ranges (IQRs) instead of means with standard deviations, and no validated method currently exists to reliably convert medians and IQRs into means and standard deviations, as noted by Wan et al. (14). Ultimately, 13 studies were included in this study (9–11, 38–47) (Figure 1).

**Figure 1:**
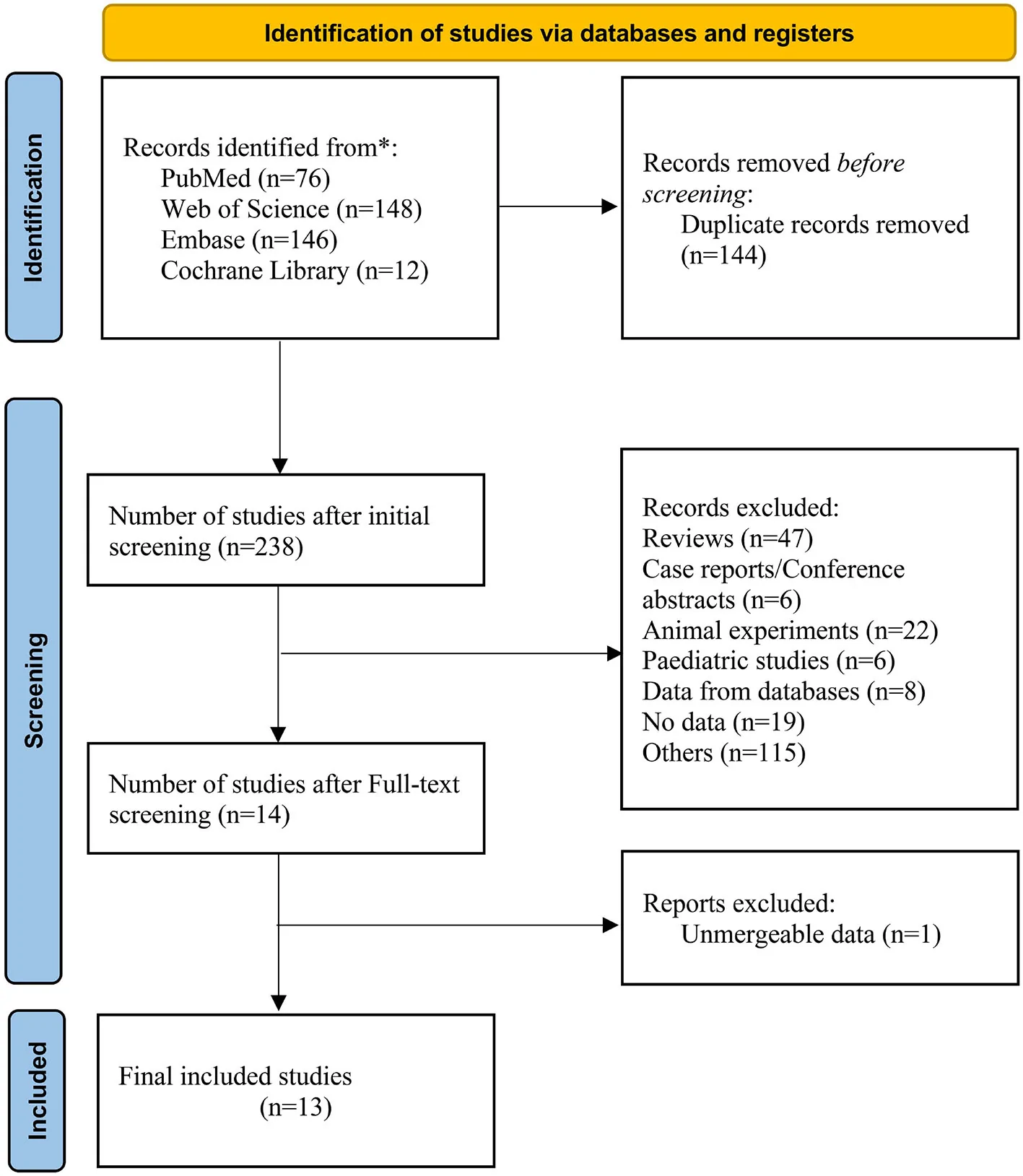
Flow diagram of literature screening. ^*^indicates that these literature records are retrieved from electronic databases.

### 3.2 Characteristics of included studies and risk of bias assessment

Among the included studies, seven investigated blood biomarkers, while six examined CSF biomarkers. A total of 804 participants were included across all studies. Patient characteristics and blood biomarker levels were summarized in Tables 1, 2, whereas CSF biomarker levels were summarized in Tables 3, 4. Risk of bias was assessed using the ROBINS-E tool. Most studies did not report results related to deviations from intended interventions. The majority demonstrated a moderate risk of bias in confounding and selection domains, while risks of bias related to classification of interventions, outcome measurement, and selective reporting were generally low (Figure 2).

**Table 1:** Characteristics of patients in studies reporting blood biomarkers.

Study	Group	Sample size	Age	F	M	BMI (kg/m^2^)	TST (min)	SE (%)	AHI (events/h)	Mean_SaO_2_ (%)	SaO_2_ nadir (%)	ODI (events/h)
Bu et al. (38)	OSA	45	44.31 ± 9.96	33	12	26.84 ± 2.52	397.30 ± 43.84	84.56 ± 9.86	32.68 ± 23.34	90.93 ± 2.9	80.60 ± 7.17	26.45 ± 17.76
	Control	49	42.92 ± 9.62	34	15	26.07 ± 2.61	404.9 ± 51.67	85.24 ± 7.32	2.26 ± 1.35	95.61 ± 1.17	91.20 ± 1.68	2.17 ± 1.27
Motamedi et al. (40)	Mild	22	34.0 ± 8.19	21	1	30.5 ± 4.80	419.02 ± 49.6	92.63 ± 5.5	8.56 ± 2.5	86.38 ± 5.4	/	/
	Moderate to severe	28	35.6 ± 7.77	28	0	31.1 ± 3.75	392.46 ± 72.3	89.83 ± 7.3	26.09 ± 21.3	82.48 ± 7.4	/	/
	Control	24	30.9 ± 7.77	22	2	28.6 ± 3.81	415.08 ± 49.4	92.68 ± 5.4	2.12 ± 1.3	90.00 ± 3.7	/	/
Przybylska-Kuć et al. (42)	Mild to moderate	38	52.2 ± 10.1	31	7	32.1 ± 6.8	/	/	15.7 ± 7.2	92.2 ± 1.9	79.1 ± 7.2	20.2 ± 14.5
	Severe	43	54.4 ± 10.4	37	6	35 ± 6.3	/	/	54.1 ± 17.7	88.9 ± 3.9	65.3 ± 14.5	59.5 ± 20.7
	Control	31	46.1 ± 14.1	17	14	31.4 ± 6.1	/	/	2 ± 1.7	92.6 ± 1.8	85.7 ± 4.3	5.3 ± 6.1
Kong (10)	OSA	35	39.18 ± 9.22	30	5	25.65 ± 2.78	/	/	63.92 ± 23.65	91.43 ± 3.4	71.91 ± 13.71	64.57 ± 26.82
	Control	16	42.43 ± 9.79	14	2	23.02 ± 2.70	/	/	2.62 ± 1.93	95.29 ± 0.99	87.25 ± 6.93	2.81 ± 2.25
Bhuniya et al. (41)	OSA	46	44.1 ± 12.4	44	2	33.2 ± 8.9	/	/	/	/	/	/
	Mild	10	/	/	/	/	344.0 ± 186.5	72.3 ± 27.7	5.0 ± 2.4	93.0 ± 4.0	/	12.9 ± 14.2
	Moderate	9	/	/	/	/	257 ± 239	74.9 ± 28.3	15.8 ± 9.8	96.0 ± 2.0	/	14.7 ± 11.1
	Severe	27	/	/	/	/	425.5 ± 181.2	82.8 ± 26.8	51.2 ± 28.9	93.0 ± 5.5	/	52.5 ± 36.9
	Control	30	29.7 ± 5.9	25	5	24 ± 1.5	/	/	/	/	/	/
Chen et al. (44)	OSA	30	41.93 ± 1.65	27	3	26.18 ± 0.52	/	/	41.93 ± 4.33	95.00 ± 0.27	/	32.75 ± 4.22
	Control	34	43.21 ± 2.25	17	17	24.83 ± 0.54	/	/	2.68 ± 0.29	97.06 ± 0.19	/	0.91 ± 0.21
Pai et al. (44)	OSA	20	40.3 ± 8.7		20	/	/	/	/	/	/	/
	Control	37	63.1 ± 12.4	14	23	/	/	/	/	/	/	/

Data are presented as mean ± standard deviation unless otherwise specified;

‘/' indicates not reported, likewise hereafter.

**Table 2:** Detailed data on blood biomarkers in included studies.

Study	Group	Aβ40 (pg/ml)	Aβ42 (pg/ml)	Aβ42/40	T-Aβ (pg/ml)	T-tau (pg/ml)	P-tau (pg/ml)
Bu et al. (38)	OSA	107.54 ± 65.77	86.37 ± 61.14	/	193.91 ± 122.41	45.40 ± 34.24	42.03 ± 21.58
	Control	71.58 ± 34.70	50.91 ± 26.93	/	125.35 ± 66.67	40.49 ± 24.61	30.56 ± 17.30
Motamedi et al. (40)	Mild	/	/	/	/	2.95 ± 2.02	/
	Moderate to severe	/	/	/	/	5.39 ± 2.57	/
	Control	/	/	/	/	2.48 ± 1.94	/
Przybylska-Kuć et al. (42)	Mild to moderate	159.4	85.9 ± 60.1	/	/	/	/
	Severe	191.1	86.9 ± 53.3	/	/	/	/
	Control	76.9	79.5 ± 54.1	/	/	/	/
Kong (10)	OSA	29.24 ± 32.52	0.23 ± 0.17	/	/	11.88 ± 7.05	26.31 ± 14.41
	Control	13.18 ± 10.78	0.26 ± 0.22	/	/	7.64 ± 4.17	17.34 ± 9.12
Bhuniya et al. (43)	OSA	121.0 ± 51.0	105.6 ± 119.5	1.0 ± 1.0	223.1 ± 157.8	168.5 ± 85.9	8.1 ± 10.4
	Mild	137.3 ± 36.1	87.9 ± 89.5	0.6 ± 0.5	225.1 ± 120.3	189.4 ± 84.3	8.9 ± 4.1
	Moderate	120.3 ± 40.4	106.8 ± 119.3	1.1 ± 1.0	226 ± 166.2	189.7 ± 47.9	4.0 ± 6.7
	Severe	113.2 ± 68.6	111.7 ± 119.2	1.1 ± 1.0	219.3 ± 144.7	159.4 ± 85.7	9.4 ± 13.1
	Control	78.3 ± 89.1	18.6 ± 72.4	0.4 ± 1.1	137.8 ± 106.2	10.9 ± 101.5	10.2 ± 13.4
Chen et al. (44)	OSA	53.00 ± 0.90	16.17 ± 0.12	0.30 ± 0.04	/	21.43 ± 0.55	/
	Control	54.61 ± 1.25	15.37 ± 0.26	0.29 ± 0.08	/	18.27 ± 0.85	/
Pai et al. (47)	OSA	/	15.97 ± 0.54	/	/	20.17 ± 2.77	/
	Control	/	14.26 ± 1.42	/	/	15.13 ± 3.62	/

**Table 3:** Characteristics of patients in studies reporting CSF biomarkers.

Study	Group	Sample size	Age	F	M	BMI (kg/m^2^)	TST (min)	SE (%)	AHI (events/h)	Mean_SaO_2_ (%)	SaO_2_ nadir (%)	ODI (events/h)
Ju et al. (52)	OSA	10	56.4 ± 4.0	7	3	29.6 ± 3.7	356 ± 70	75 ± 11	28.6 ± 12.5	/	80 ± 11.1	/
	Control	31	53.2 ± 5.7	16	15	26.6 ± 3.9	391 ± 44	79 ± 9	1.6 ± 1.4	/	89 ± 2.6	/
Liguori et al. (9)	OSA	25	67.96 ± 7.92	17	8	28.92 ± 2.38	337.95 ± 94.57	73.45 ± 14.94	36.34 ± 11.42	92.37 ± 1.97	78.55 ± 6.02	32.08 ± 12.78
	Control	15	66.33 ± 8.92	9	6	27.2 ± 1.90	364.13 ± 50.61	87.79 ± 4.65	5.21 ± 4.38	95.87 ± 1.19	90.33 ± 3.66	4.39 ± 3.99
Liguori et al. (41)	OSA	20	58.75 ± 3.53	14	6	27.63 ± 2.65	335.2 ± 84.36	69.58 ± 14.4	33.7 ± 19.01	/	/	/
	Control	15	63.8 ± 8.46	8	7	24.59 ± 1.89	298.25 ± 67.37	88.22 ± 4.63	7.3 ± 2.16	/	/	/
Díaz-Román et al. (11)	Total	57	66.19 ± 7.13	26	31	26.61 ± 4.01	285.28 ± 68.98	68.28 ± 16.54	25.56 ± 22.35	93.82 ± 1.71	/	/
Fernandes et al. (41)	OSA	20	58.75 ± 3.53	14	8	/	335.2 ± 84.36	69.58 ± 14.4	33.7 ± 19.01	/	/	/
	Control	15	63.8 ± 8.46	6	7	/	298.25 ± 67.37	88.22 ± 4.63	/	/	/	/
Fernandes et al (42)	OSA	34	68.44 ± 9.98	27	7	/	347 ± 71.86	77.53 ± 12.34	44.52 ± 20.46	94.37 ± 1.85	79.95 ± 5.94	34.8 ± 16.69
	Control	34	69.02 ± 6.08	27	7	/	/	/	/	/	/	/

**Table 4:** Detailed data on CSF biomarkers in included studies.

Study	Group	Aβ40 (pg/ml)	Aβ42 (pg/ml)	Aβ42/A β40	T-tau (pg/ml)	P-tau (pg/ml)
Ju et al. (39)	OSA	7,900 ± 2,483	717 ± 143	0.097 ± 0.029	167 ± 80	32 ± 13
	Control	11,208 ± 3.420	914 ± 224	0.084 ± 0.015	221 ± 92	42 ± 14
Liguori et al. (9)	OSA	/	484.00 ± 159.90	/	296.21 ± 227.69	47.67 ± 33.35
	Control	/	888.40 ± 147.88	/	222.33 ± 67.38	40.47 ± 10.33
Liguori et al. (41)	OSA	7,090.45 ± 2,739.1	622.05 ± 270.43	0.09 ± 0.04	205.35 ± 98.59	39.1 ± 14.88
	Control	10,561.6 ± 2,688.47	889.4 ± 423.16	0.11 ± 0.04	205.35 ± 98.59	38.3 ± 6.84
Díaz-Román et al. (11)	Mild	/	1295.20 ± 519.97	/	324.00 ± 206.76	53.30 ± 18.28
	Moderate	/	833.33 ± 311.96	/	388.83 ± 283.11	63.83 ± 40.64
	Severe	/	862.93 ± 277.36	/	662.33 ± 567.55	84.73 ± 58.81
	Control	/	1,182.90 ± 456.78	/	327.30 ± 337.53	50.60 ± 31.71
Fernandes et al. (45)	OSA	/	622.05 ± 270.43	/	205.35 ± 98.59	39.1 ± 14.88
	Control	/	889.4 ± 323.16	/	176.2 ± 89.27	38.3 ± 6.84
Fernandes et al. (46)	OSA	/	622.78 ± 211.96	/	335.30 ± 335.39	62.34 ± 49.37
	Control	/	921.96 ± 69.55	/	252.89 ± 43.26	32.75 ± 5.21

**Figure 2:**
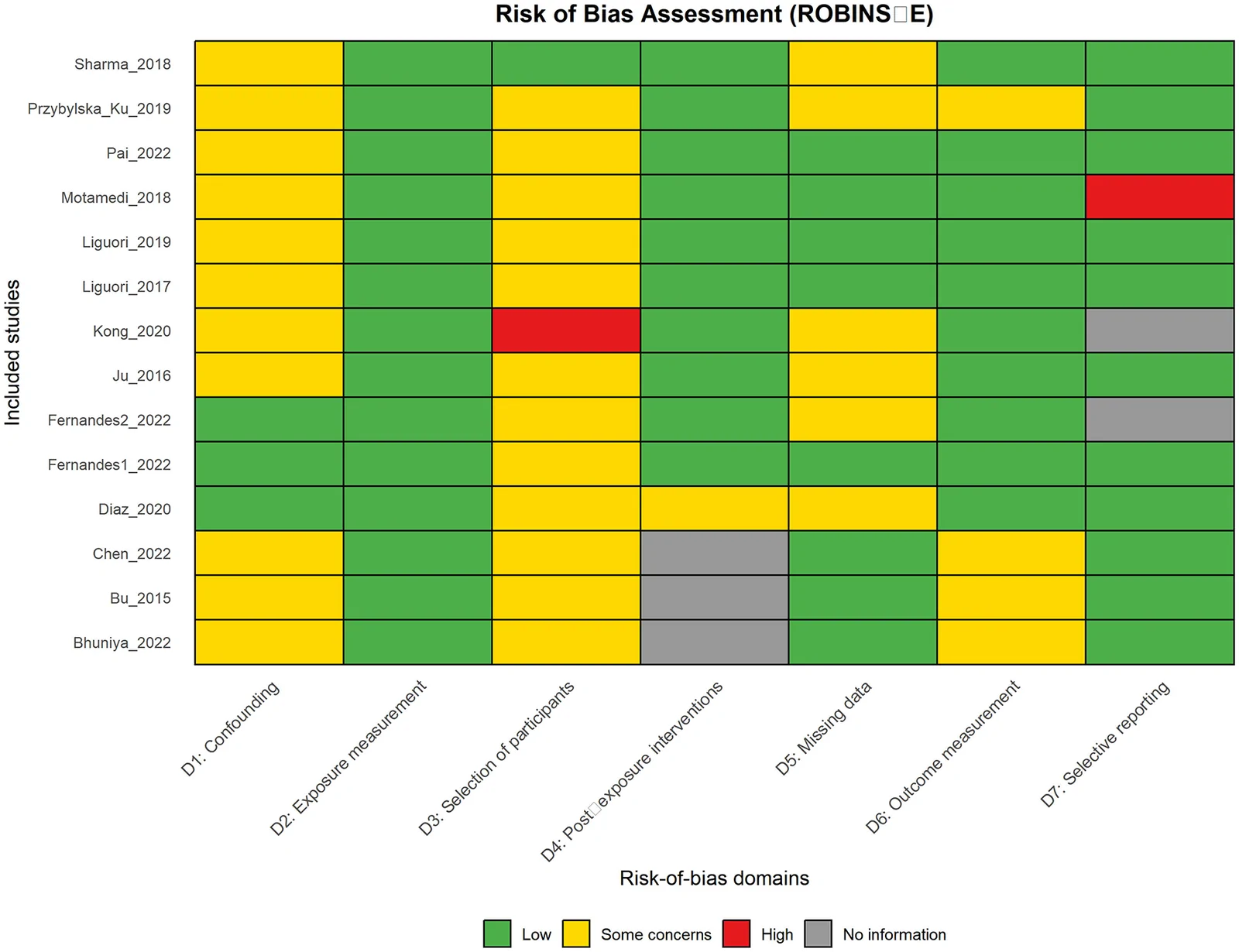
Risk of bias assessment (H: high risk; M: moderate risk; L: low risk; N: not reported).

### 3.3 Meta-analysis results

#### 3.3.1 Blood neurodegenerative biomarkers

Meta-analysis results are presented in Figure 3 and Table 5. Compared with controls, patients with OSA showed significantly elevated levels of *T*-Aβ (SMD = 0.66, 95% CI [0.34, 0.97], *I*^2^ = 0%, *p* < 0.01) and *T*-tau (SMD = 1.48, 95% CI [0.33, 2.62], 95% PI [**–**2.41, 5.36], *I*^2^ = 93.6%, *p* = 0.011). Notably, the 95% PIs of both biomarkers crossed the null value despite statistically significant pooled effects. In contrast, no significant differences were observed between OSA patients and controls for Aβ40, Aβ42, Aβ42/Aβ40 ratio, or *P*-tau. Funnel plots for publication bias are shown in Figure 4.

**Figure 3:**
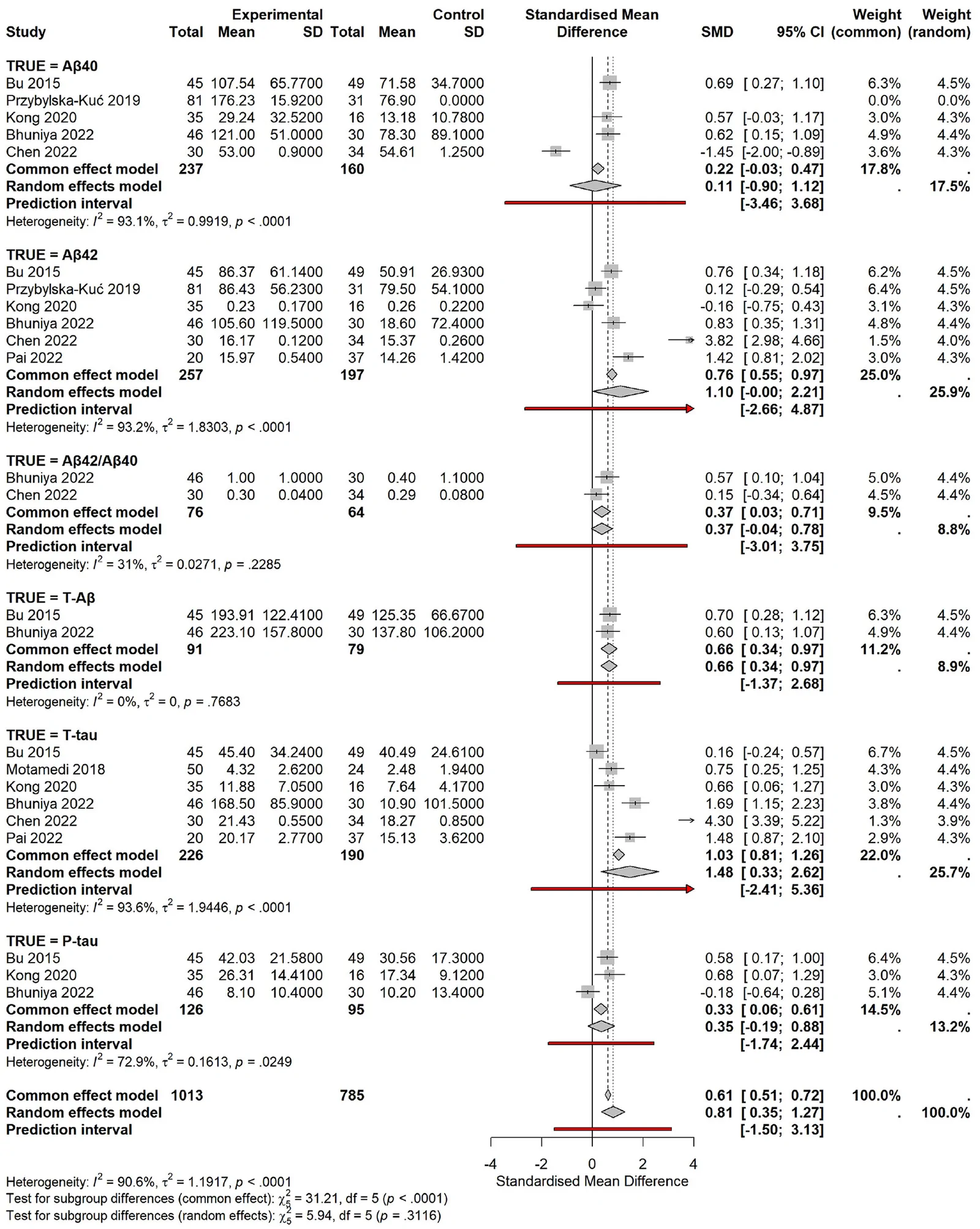
Meta-analysis of blood neurodegenerative biomarkers.

**Table 5:** Meta-analysis results of biomarkers based on random-effects model.

Biomarkers	No of studies	Sample size	SMD (95% CI)	*P-value*
**Blood (pg/ml)**				
Aβ40	4	397	0.11 [−0.90; 1.12]	0.826
Aβ42	6	454	1.10 [−0.00; 2.21]	0.0506
Aβ42/40	2	140	0.37 [−0.04; 0.78]	0.0772
T-Aβ	2	170	0.66 [ 0.34; 0.97]	< 0.001
T-tau	6	416	1.48 [ 0.33; 2.62]	0.011
P-tau	3	221	0.35 [−0.19; 0.88]	0.203
**CSF (pg/ml)**				
Aβ40	2	76	−1.13 [−1.65; −0.60]	< 0.001
Aβ42	6	289	−1.23 [−1.84; −0.63]	< 0.001
Aβ42/40	2	76	0.08 [−1.05; 1.21]	0.889
T-tau	6	289	0.23 [−0.02; 0.49]	0.0674
P-tau	6	289	0.20 [−0.21; 0.61]	0.341

**Figure 4:**
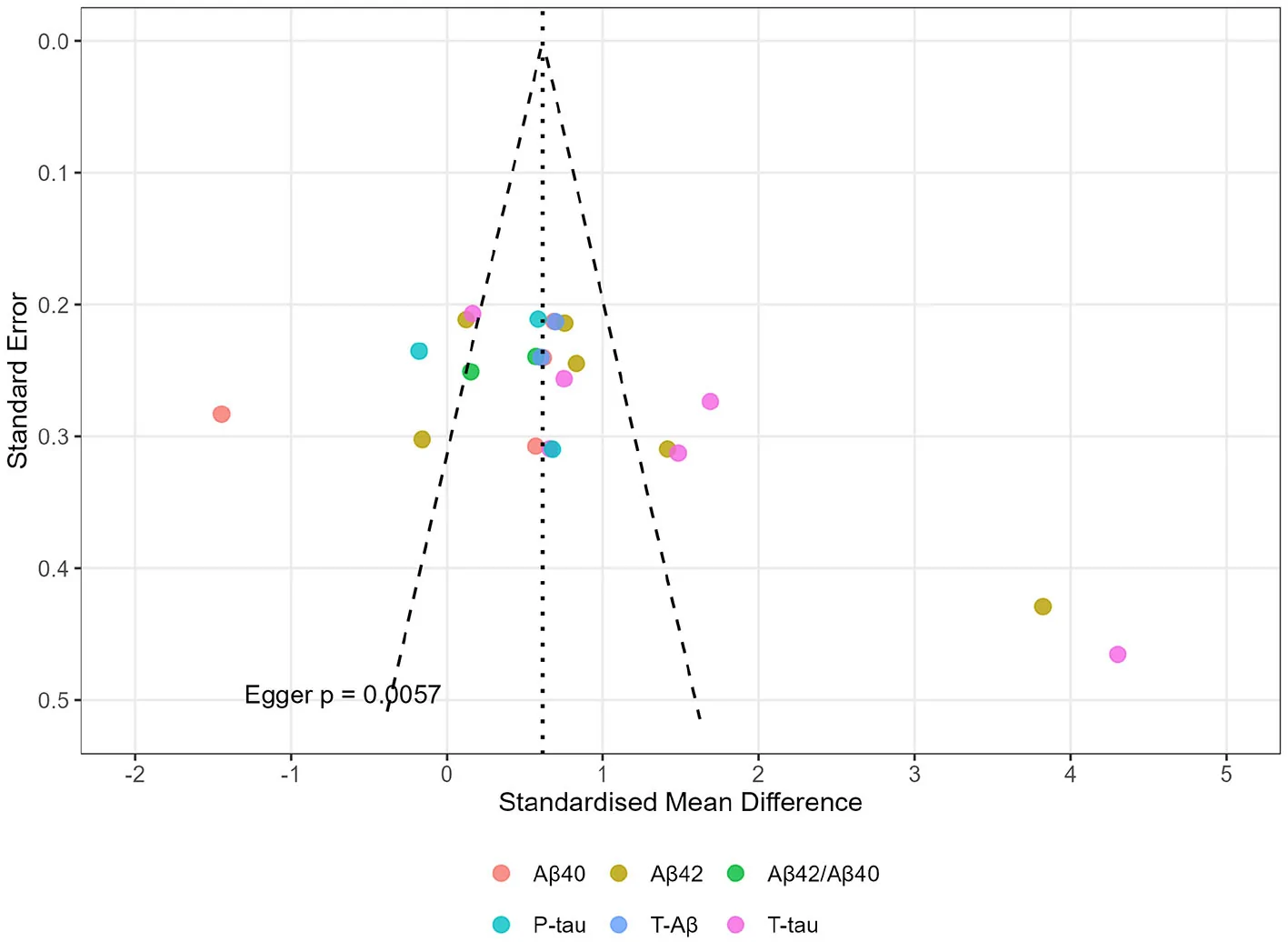
Funnel plot for blood neurodegenerative biomarkers.

High heterogeneity was observed for Aβ40 (*I*^2^ = 93.1%), Aβ42 (*I*^2^ = 93.2%), *T*-tau (*I*^2^ = 93.6%), and *P*-tau (*I*^2^ = 72.9%). Funnel plot inspection suggested potential asymmetry for *T*-tau, warranting cautious interpretation of the pooled estimate. To further investigate the sources of this heterogeneity and assess the robustness of the pooled estimates, we performed leave-one-out sensitivity analyses. For blood biomarkers, regarding Aβ40, excluding the study by Chen et al. (44) reduced the *I*^2^ to 0%, and the pooled effect estimate not only reversed direction but also became statistically significant, indicating that this study was the primary source of the extreme heterogeneity. For blood Aβ42, after excluding Chen et al. (44) the *I*^2^ decreased but remained substantially high at 79.9% (Figure 5). Although the pooled estimate became statistically significant (SMD = 0.59, 95% CI [0.07, 1.11], *p* = 0.0006), this finding should be interpreted as exploratory due to the persistent substantial heterogeneity, which precludes a definitive conclusion regarding blood Aβ42 elevation. For blood *T*-tau, even after excluding Chen et al. (44) the *I*^2^ remained as high as 84.0% (Figure 6), suggesting that both heterogeneity and bias compounded the pooled results for *T*-tau.

**Figure 5:**
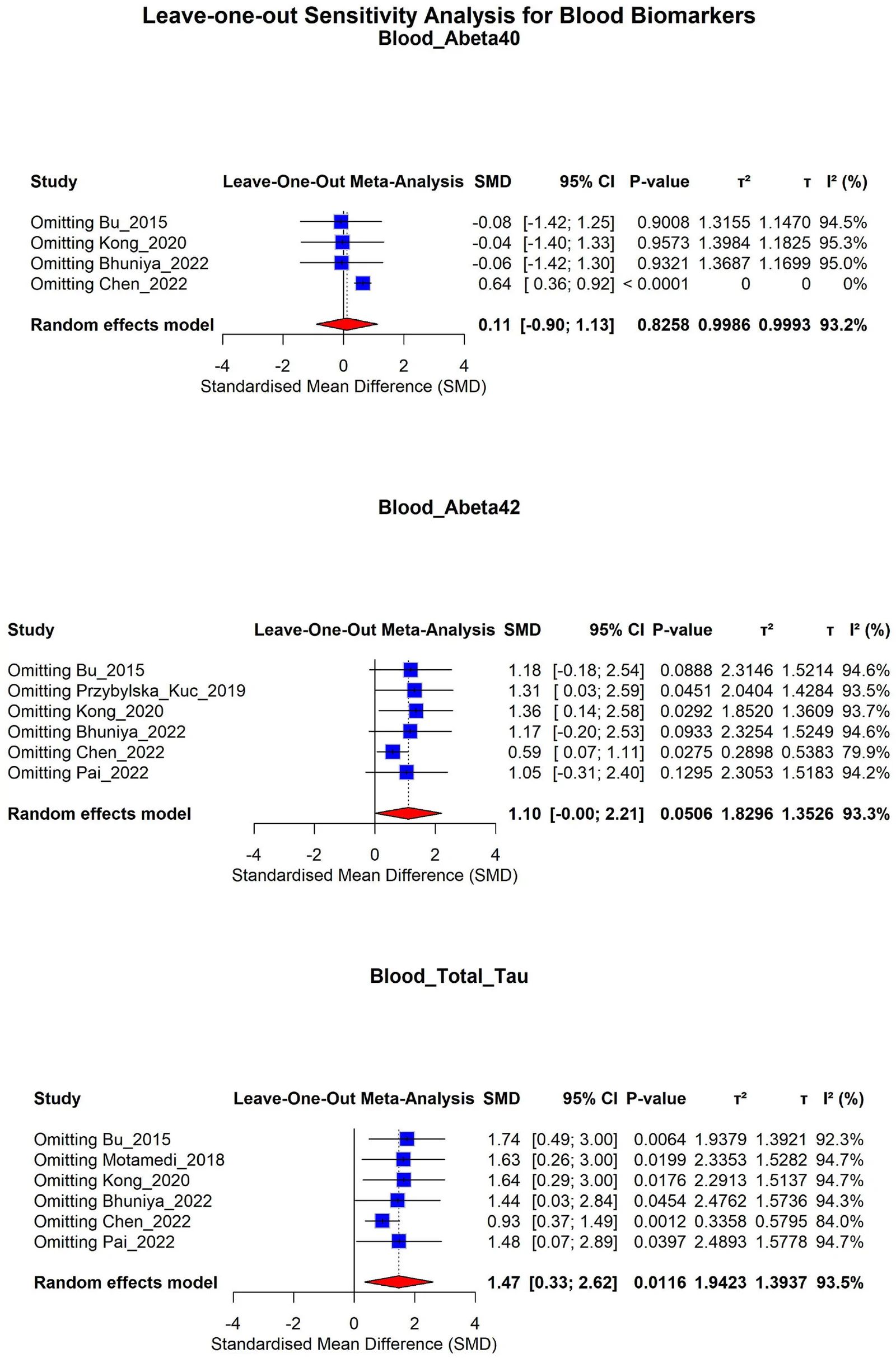
Leave-one-out sensitivity analysis for blood biomarkers.

**Figure 6:**
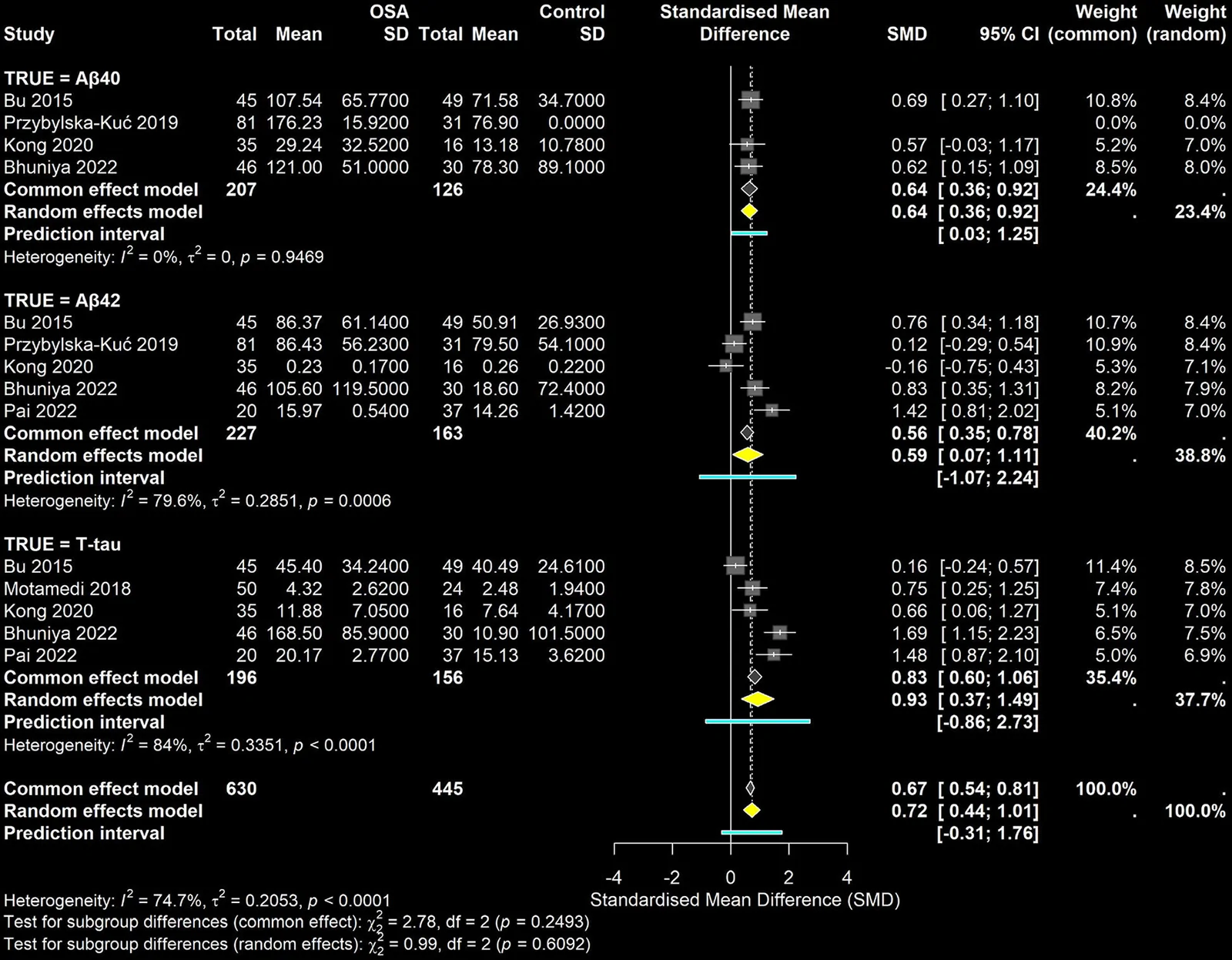
Meta-analysis of blood biomarkers levels (after excluding high-heterogeneity studies).

#### 3.3.2 Cerebrospinal fluid neurodegenerative biomarkers

Meta-analysis results are presented in Figure 7 and Table 5. Compared with controls, patients with OSA had significantly lower levels of CSF Aβ40 (SMD = **–**1.13, 95% CI [**–**1.65, **–**0.60], *I*^2^ = 0%, *p* < 0.01) and Aβ42 (SMD = **–**1.23, 95% CI [**–**1.84, **–**0.63], 95% PI [**–**3.16, 0.69], *I*^2^ = 77.3%, *p* < 0.01). The wide 95% prediction interval crossing the null for Aβ42 suggested inconsistent effect sizes across individual trials despite the significant pooled overall effect. However, no significant differences were observed for Aβ42/Aβ40 ratio, *T*-tau, or *P*-tau. Funnel plots for publication bias are shown in Figure 8. Moderate-to-high heterogeneity was detected for Aβ42 (*I*^2^ = 77.3%), Aβ42/Aβ40 ratio (*I*^2^ = 80.6%), and *P*-tau (*I*^2^ = 61.3%). Funnel plot inspection suggested potential asymmetry for *P*-tau, warranting cautious interpretation (Figure 8).

**Figure 7:**
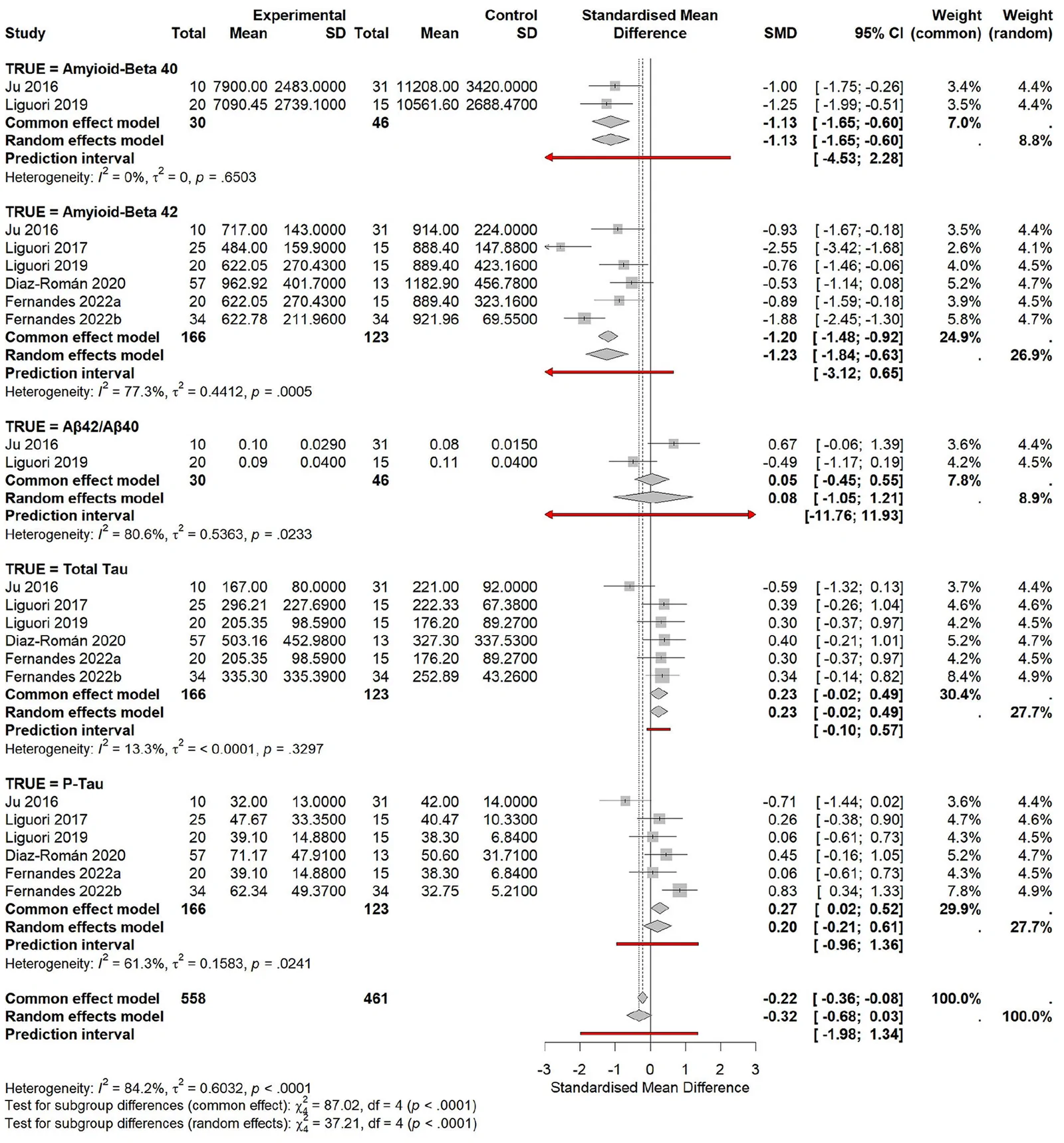
Meta-analysis of CSF neurodegenerative biomarkers.

**Figure 8:**
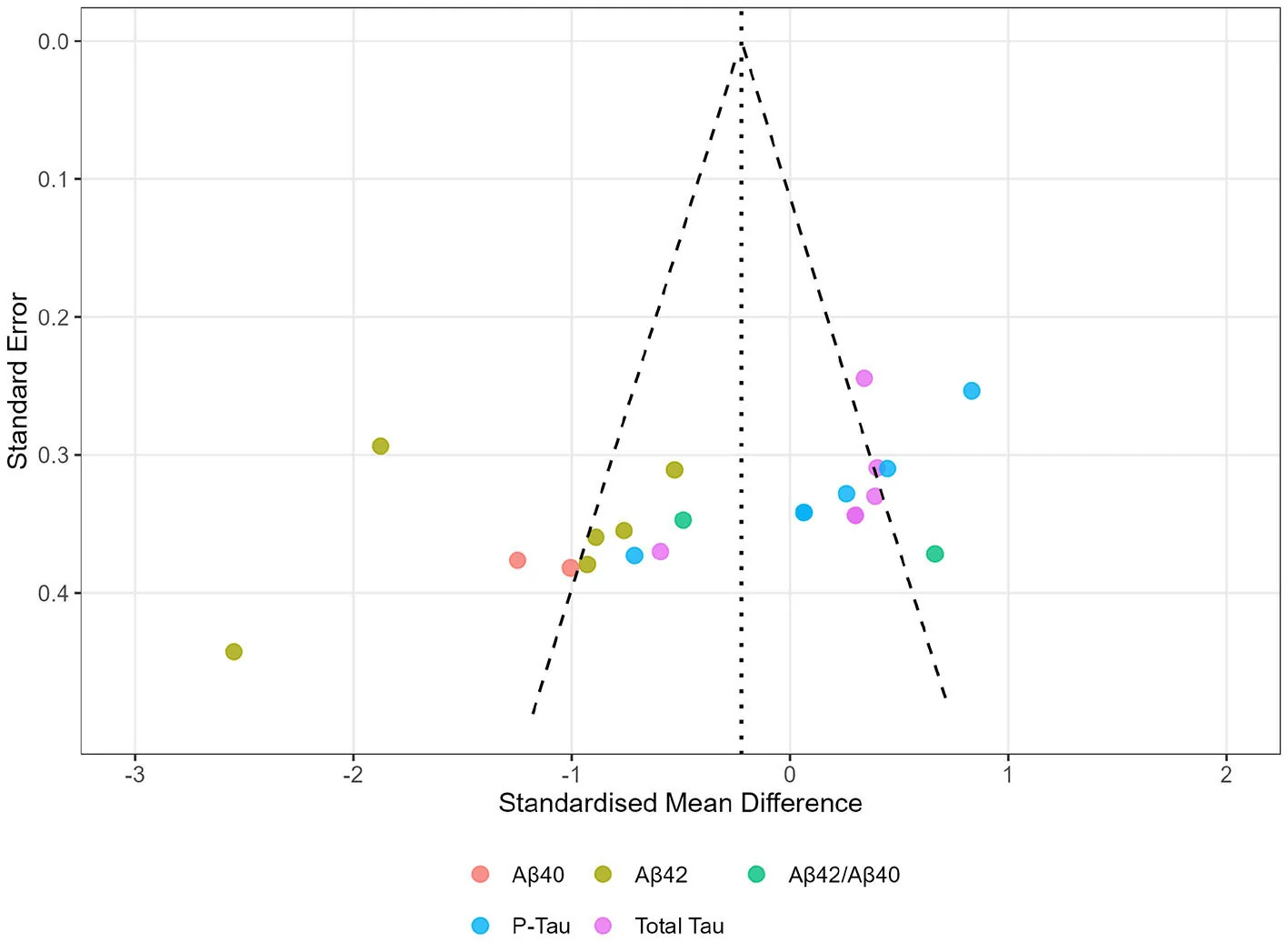
Funnel plot for CSF neurodegenerative biomarkers.

Regarding cerebrospinal fluid (CSF) biomarkers, CSF Aβ42 showed considerable heterogeneity (*I*^2^ = 77.3%). Excluding the study by Liguori et al. (9) reduced the *I*^2^ to 65.8%, and the pooled effect remained statistically significant (SMD = **–**1.01, 95% CI [**–**1.50, **–**0.52]). For CSF *P*-tau, although the primary meta-analysis did not reach statistical significance (SMD = 0.20, 95% CI [**–**0.21, 0.61], *P* = 0.3419), the leave-one-out analysis revealed extreme instability of the results. Excluding Ju et al. (39) reversed the effect estimate and rendered it statistically significant (SMD = 0.38, 95% CI [0.06, 0.70], *P* = 0.0197), with the *I*^2^ decreasing substantially from 61.3 to 21.4%. This suggests that this single study pre-dominantly drove the current negative conclusion regarding CSF *P*-tau. Sequentially excluding the remaining studies one by one reduced heterogeneity; however, no statistically significant differences were observed. The leave-one-out sensitivity analysis and forest plots are shown in Figures 9, 10.

**Figure 9:**
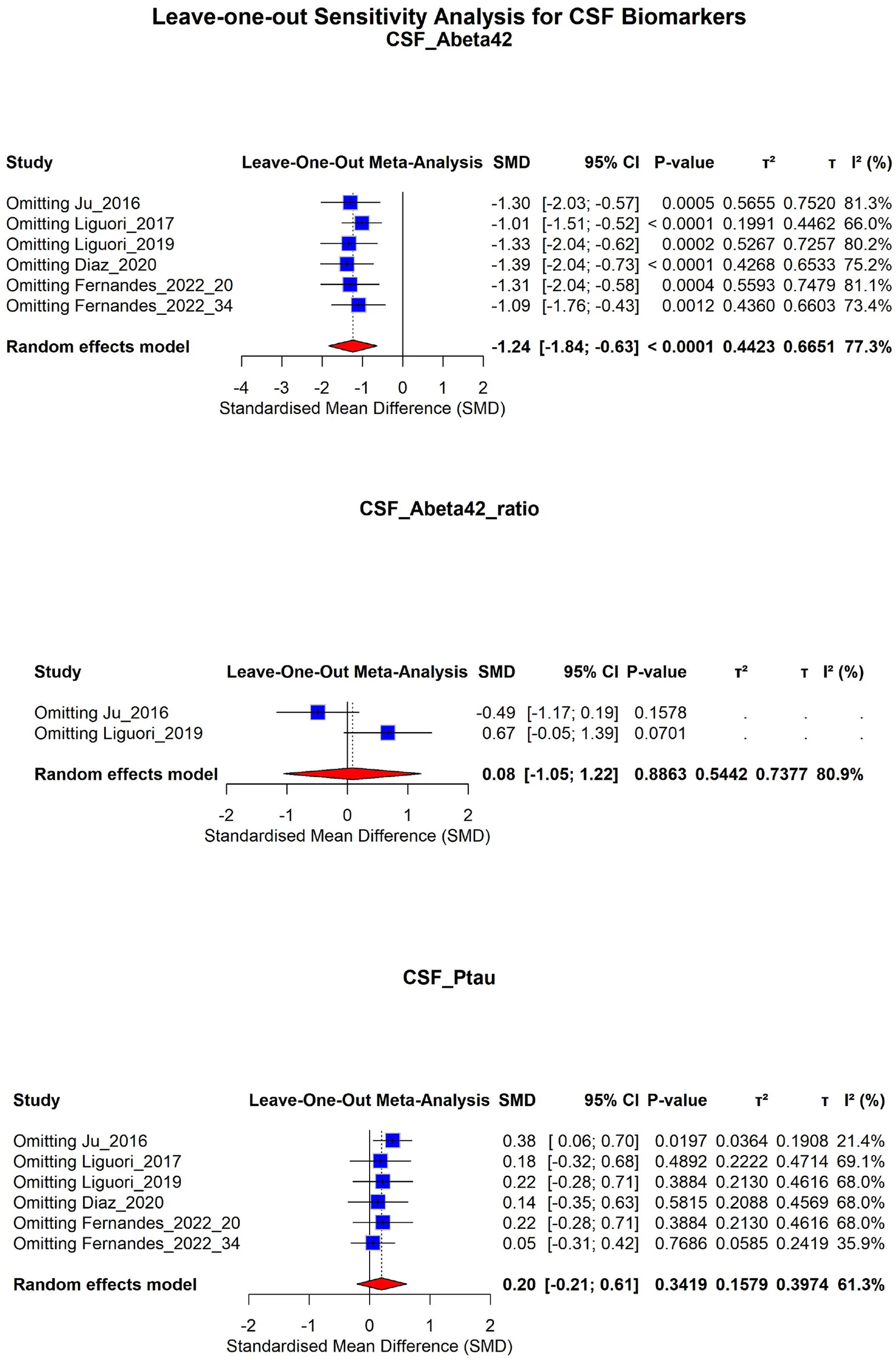
Leave-one-out sensitivity analysis for CSF biomarkers.

**Figure 10:**
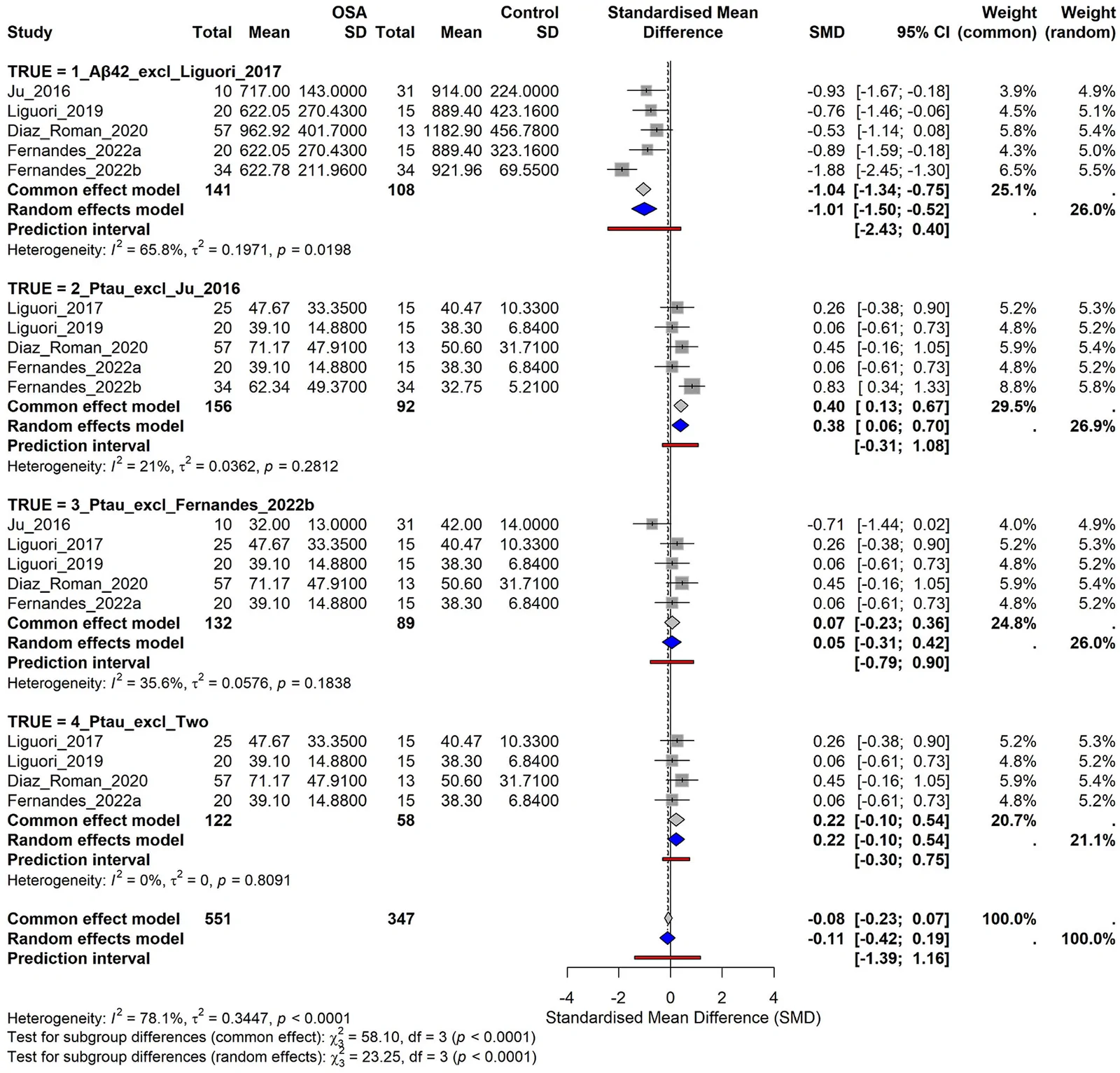
Meta-analysis of CSF biomarkers levels (after excluding high-heterogeneity studies).

### 3.4 Meta-regression

We conducted meta-regression analyses to examine the effects of age, BMI, AHI, Mean_SaO_2_, and ODI on blood biomarkers, as well as the effects of age, AHI, TST, and SE on CSF biomarkers (Table 6). For CSF biomarkers, age significantly influenced CSF *T*-tau (β = 0.04, 95% CI [0.01, 0.07], *p* = 0.009) and CSF *P*-tau (β = 0.07, 95% CI [0.02, 0.11], *p* = 0.012), explaining 100% and 76.46% of the between-study variance, respectively. No other covariates—including AHI, TST, SE, BMI, or oxygenation indices—showed significant effects on any blood or CSF biomarker (*p* > 0.05). Given the limited number of studies per covariate (ranging from 2 to 6), these meta-regression findings should be interpreted as exploratory and hypothesis-generating rather than confirmatory. They were insufficiently powered to adjust for multiple confounders simultaneously.

**Table 6:** Univariable meta-regression results.

Sample	Biomarker	Characteristics	Group	β	95%CI	SE	*Z*	*p*-value	*R*^2^%	*I*^2^_res%
blood	Aβ42	Age	OSA	−0.08	[−0.33, 0.18]	0.13	−0.58	0.49	0	96.60
			Control	0.01	[−0.11, 0.14]	0.06	0.22	0.78	0	97.09
		BMI	OSA	−0.13	[−0.55, 0.29]	0.21	−0.61	0.82	0	97.52
			Control	−0.10	[−0.62, 0.43]	0.27	−0.36	0.66	0	97.61
		AHI	OSA	−0.03	[−0.17, 0.10]	0.07	−0.46	0.70	0	97.74
		Mean_SaO2	OSA	0.74	[0.29, 1.19]	0.23	3.23	0.06	74.01	90.29
		ODI	OSA	−0.05	[−0.15, 0.05]	0.05	−1.03	0.43	0	97.11
	T-tau	Age	OSA	0.07	[−0.32, 0.47]	0.2	0.37	0.7	0	96.51
			Control	0.01	[−0.11, 0.13]	0.06	0.17	0.87	0	96.91
		BMI	OSA	−0.06	[−0.61, 0.49]	0.28	−0.2	0.93	0	97.66
			Control	−0.17	[−1.00, 0.66]	0.42	−0.4	0.68	0	97.60
		AHI	OSA	0.01	[−0.13, 0.15]	0.07	0.15	0.88	0	98.47
		Mean_SaO2	OSA	0.26	[−0.20, 0.72]	0.23	1.1	0.46	5.34	97.45
CSF	Aβ42	Age	OSA	−0.09	[−0.20.0.02]	0.06	−1.56	0.28	26.32	72.98
			Control	−0.05	[−0.18, 0.07]	0.06	−0.83	0.48	0	80.33
		AHI	OSA	−0.08	[−0.15,−0.01]	0.04	−2.13	0.10	55.8	60.23
		TST	OSA	−0.01	[−0.04, 0.01]	0.01	−1	0.41	0.7	77.76
			Control	−0.01	[−0.02.0.01]	0.01	−1.15	0.28	9.03	75.68
		SE	OSA	−0.13	[−0.28.0.01]	0.07	−1.88	0.15	46.47	65.08
			Control	−0.04	[−0.13, 0.05]	0.04	−0.93	0.35	0	77.43
	T-tau	Age	OSA	0.04	[−0.01, 0.09]	0.03	1.53	0.02	10.98	0.00
			Control	0.06	[0.01, 0.11]	0.03	2.22	0.06	100	0.00
		AHI	OSA	0.02	[−0.03, 0.06]	0.02	0.7	0.44	0	16.88
		TST	OSA	−0.01	[−0.02, 0.01]	0.01	−1.05	0.22	0	6.25
			Control	−0.01	[−0.01, 0.00]	0	−1.42	0.12	99.99	0.00
		SE	OSA	−0.02	[−0.11, 0.06]	0.04	−0.56	0.74	0	24.22
			Control	0	[−0.05, 0.05]	0.03	0.06	1.00	0	44.97
	P-tau	Age	OSA	0.08	[0.03, 0.14]	0.03	3.09	0.01	100	0.00
			Control	0.1	[0.04, 0.15]	0.03	3.47	0.01	100	0.00
		AHI	OSA	0.04	[−0.01, 0.10]	0.03	1.46	0.18	31.54	49.61
		TST	OSA	−0.01	[−0.03, 0.01]	0.01	−0.69	0.45	0	65.91
			Control	−0.01	[−0.01, 0.00]	0	−1.6	0.18	77.17	10.57
		SE	OSA	0.02	[−0.11, 0.15]	0.07	0.28	0.82	0	66.45
			Control	−0.01	[−0.06, 0.05]	0.03	−0.28	0.78	0	49.79

### 3.5 GRADE certainty of evidence

We evaluated the overall certainty of evidence for all biomarker outcomes that underwent meta-analysis using the GRADE framework. Table 7 presents the full GRADE assessments for each biomarker, including primary and all secondary outcomes. The ratings ranged from moderate to very low across the different biomarkers, with imprecision and inconsistency being the most common reasons for downgrading.

**Table 7:** Summary of findings and GRADE certainty of evidence.

Biomarker	Number of studies (participants)	SMD (95% CI)	*I* ^2^	Risk of bias	Inconsistency	Indirectness	Imprecision	Publication bias	Overall certainty
**Blood**									
T-Aβ	2 (170)	0.66 (0.34, 0.97)	0%	No concern	No concern	No concern	Serious①	No concern	⊕⊕⊖⊖ LOW
T-tau	6 (416)	1.48 (0.33, 2.62)	93.6%	No concern	Very serious②	No concern	Serious①	Not assessed③	⊕⊖⊖⊖ VERY LOW
Aβ40	4 (397)	0.11 (−0.90, 1.12)	93.1%	No serious	Very serious④	No serious	Serious⑤	No serious	⊕⊖⊖⊖ VERY LOW
Aβ42	6 (454)	1.10 (−0.00, 2.21)	93.2%	No serious	Very serious④	No serious	Serious⑤	No serious	⊕⊖⊖⊖ VERY LOW
Aβ42/40	2 (140)	0.37 (−0.04, 0.78)	0%	No serious	No serious	No serious	Very serious⑥	Not assessed③	⊕⊕⊖⊖ LOW
P-tau	3 (221)	0.35 (−0.19, 0.88)	72.9%	No serious	Serious⑦	No serious	Serious⑤	No serious	⊕⊕⊖⊖ LOW
**CSF**									
Aβ40	2 (76)	−1.13 (−1.65, −0.60)	0%	No serious	No serious	No serious	No serious	Not assessed③	⊕⊕⊕⊖ MODERATE
Aβ42	6 (289)	−1.23 (−1.84, −0.63)	77.3%	No serious	Serious⑦	No serious	Serious②	Not assessed③	⊕⊕⊖⊖ LOW
Aβ42/40	2 (76)	0.08 (−1.05, 1.21)	80.6%	No serious	Serious⑦	No serious	Very serious⑥	Not assessed③	⊕⊖⊖⊖ VERY LOW
T-tau	6 (289)	0.23 (−0.02, 0.49)	0%	No serious	No serious	No serious	Serious⑤	Not assessed③	MODERATE
P-tau	6(289)	0.20 (-0.21, 0.61)	61.3%	No serious	Serious⑦	No serious	Serious⑤	Serious⑨	VERY LOW

For blood biomarkers, the overall certainty ranged from low to very low. Blood *T*-Aβ (SMD = 0.66, 95% CI [0.34, 0.97], *I*^2^ = 0%) was rated as low certainty (⊕⊕⊖⊖), downgraded by one level due to serious imprecision stemming from the limited pooled sample size (*n* = 170), with no serious concerns in other domains. Blood *T*-tau (SMD = 1.48, *I*^2^ = 93.6%) was rated as very low certainty (⊕⊖⊖⊖) driven by very serious inconsistency (prediction interval crossing the null) and serious imprecision, with additional concerns about publication bias based on funnel plot asymmetry. Similarly, blood Aβ40 (SMD = 0.11, *I*^2^ = 93.1%) and blood Aβ42 (SMD = 1.10, *I*^2^ = 93.2%) were both rated as very low certainty (⊕⊖⊖⊖) due to very serious inconsistency and serious imprecision (wide confidence intervals crossing the null). Blood Aβ42/Aβ40 ratio (SMD = 0.37, 95% CI [−0.04, 0.78], *I*^2^ = 0%) was rated as low certainty (⊕⊕⊖⊖), with very serious imprecision because the estimate was derived from only two studies with a total sample size of 140. Blood *P*-tau (SMD = 0.35, 95% CI [−0.19, 0.88], *I*^2^ = 72.9%) was also rated as low certainty (⊕⊕⊖⊖), downgraded for serious inconsistency and serious imprecision.

For cerebrospinal fluid (CSF) biomarkers, the certainty of evidence was more variable, ranging from moderate to very low. CSF Aβ40 (SMD = −1.13, 95% CI [−1.65, −0.60], *I*^2^ = 0%) was rated as moderate certainty (⊕⊕⊕⊖), as no serious concerns were identified across any domain, with an adequate sample size and narrow confidence intervals. CSF Aβ42 (SMD = −1.23, *I*^2^ = 77.3%) was rated as low certainty (⊕⊕⊖⊖), downgraded for serious inconsistency (prediction interval approaching the null) and serious imprecision. CSF *T*-tau (SMD = 0.23, 95% CI [−0.02, 0.49], *I*^2^ = 0%) received a moderate certainty (⊕⊕⊕⊖) rating, as the only notable concern was borderline imprecision (the confidence interval touches the null); no other domains were downgraded. In contrast, CSF Aβ42/Aβ40 ratio (SMD = 0.08, 95% CI [−1.05, 1.21], *I*^2^ = 80.6%) was rated as very low certainty (⊕⊖⊖⊖) due to very serious imprecision from extremely wide confidence intervals and serious inconsistency. CSF *P*-tau (SMD = 0.20, 95% CI [−0.21, 0.61], *I*^2^ = 61.3%) was also rated as very low certainty (⊕⊖⊖⊖), downgraded for serious inconsistency, serious imprecision, and significant publication bias based on an asymmetric funnel plot.

Overall, the GRADE assessments indicate that the current evidence base provides moderate certainty for CSF Aβ40 and CSF *T*-tau, suggesting that further research may impact confidence but is unlikely to change the direction of effect. For most blood biomarkers and CSF Aβ42, the low-to-very-low certainty ratings imply that future studies are likely to alter our confidence in these estimates substantially and may even change the magnitude or direction of the observed associations. These ratings are incorporated into the interpretation of our findings throughout the Discussion section.

## 4 Discussion

### 4.1 Overview of main findings

In this meta-analysis, we included seven studies involving 528 patients that examined blood biomarkers and six studies involving 289 patients that examined CSF biomarkers. Compared with healthy controls, patients with OSA demonstrated significantly elevated levels of *T*-Aβ and *T*-tau in blood, as well as significantly reduced levels of Aβ40 and Aβ42 in CSF. However, substantial heterogeneity was observed for blood *T*-tau and CSF Aβ42 levels, and publication bias was detected for blood *T*-tau. Therefore, while these findings suggest significant differences between OSA patients and controls, they should be interpreted cautiously and validated by further research.

Before interpreting these findings, it is important to emphasize that the vast majority of included studies are cross-sectional in design. Consequently, causality cannot be inferred from the current evidence, and the biological mechanisms discussed below—including glymphatic dysfunction, hypoxia-induced APP processing, and impaired metabolite clearance—are intended as hypothetical explanations for the observed associations rather than established causal pathways (48). Reverse causality (i.e., pre-existing neurodegenerative pathology contributing to sleep-disordered breathing) and residual confounding cannot be ruled out (49).

### 4.2 Potential mechanisms linking OSA to AD-related biomarkers

The alterations in Aβ and tau proteins may be linked to abnormal sleep patterns and oxidative stress associated with OSA. During sleep deprivation, neurons increase the release of interstitial fluid (ISF) Aβ, resulting in higher Aβ levels (50). In OSA, obstructive breathing during apnea events raises intrathoracic and intracranial pressure, which abruptly reverses upon apnoea termination, leading to repetitive pressure fluctuations. These fluctuations may impede metabolite clearance from ISF into CSF, causing retention and thereby reducing CSF Aβ levels (51). Sleep fragmentation caused by OSA may also contribute to biomarker alterations, as reduced sleep efficiency has been associated with decreased CSF Aβ42 (52).

Furthermore, poor sleep quality in otherwise healthy adults at high risk of AD has been linked to CSF biomarkers of neurodegeneration (53). Beyond arousals, hypoxia and ischemia can activate amyloid precursor protein (APP) processing, stimulating Aβ production and plaque accumulation (54). An imbalance among APP processing pathways under hypoxic conditions favoring amyloidogenic β/γ-secretase activity over non-amyloidogenic α-secretase further promotes Aβ accumulation (55).

Accumulating evidence implicates the glymphatic system—a brain-wide perivascular pathway that facilitates clearance of interstitial solutes, including amyloid-β (Aβ) and tau—as a critical link between sleep and neurodegenerative processes (56). Glymphatic clearance is pre-dominantly active during slow-wave (non-rapid eye movement) sleep, when the interstitial space expands by approximately 60%, enabling efficient removal of metabolic waste products (57). In the context of OSA, glymphatic clearance may be compromised through multiple pathways. First, OSA disrupts sleep architecture via recurrent arousals and sleep fragmentation, leading to reduced slow-wave sleep and consequently impaired glymphatic function (58). Second, apneic events generate substantial negative intrathoracic pressure swings that are transmitted to the intracranial compartment, potentially causing mechanical disruption of perivascular fluid dynamics (59). Third, intermittent hypoxia may further exacerbate glymphatic dysfunction through oxidative stress and dysregulation of aquaporin-4 expression (60).

However, if OSA indeed impairs glymphatic clearance of Aβ (61), one would expect increased cerebral Aβ deposition and corresponding CSF biomarker changes. Yet the present meta-analysis found no significant alterations in CSF *P*-tau or the Aβ42/Aβ40 ratio—the biomarkers most closely reflecting core AD pathology. This apparent paradox may arise from several interconnected factors: glymphatic dysfunction may represent an early, potentially reversible alteration that precedes overt AD-type pathology, with longitudinal studies needed to determine whether sustained impairment over years eventually translates into *p*-tau and Aβ42/Aβ40 changes; compensatory upregulation of alternative clearance pathways (e.g., blood–brain barrier transport, enzymatic degradation) may partially offset glymphatic dysfunction, preventing pathological Aβ and tau accumulation (62); the observed reduction in CSF Aβ may directly reflect impaired glymphatic clearance (i.e., less Aβ flushed from brain interstitial fluid into CSF) but does not necessarily equate to increased cerebral Aβ deposition—a distinction requiring PET imaging; and the absence of *p*-tau elevation in OSA suggests that glymphatic dysfunction may affect Aβ clearance more selectively than tau pathology, or that Aβ and tau are cleared through partially distinct pathways (63).

### 4.3 Consideration of reverse causality

A pertinent question then arises: if OSA indeed impairs glymphatic clearance of Aβ, one would expect to observe increased cerebral Aβ deposition and corresponding changes in CSF biomarkers. However, as noted above, the present meta-analysis found no significant alterations in CSF *P*-tau or the Aβ42/Aβ40 ratio—the biomarkers most closely reflecting core AD pathology. This apparent paradox, together with the possibility of reverse causality, warrants careful consideration.

While the preceding discussion has primarily centered on the hypothesis that obstructive sleep apnea (OSA) drives neurodegenerative biomarker changes, the inverse causal pathway warrants equal consideration. Accumulating evidence now indicates that early, pre-clinical Alzheimer's disease pathology may itself disrupt sleep architecture and respiratory control, thereby contributing to the onset or aggravation of OSA. This reverse-causality hypothesis is buttressed by convergent findings from multiple investigative approaches (64). Neuropathological data, for instance, reveal that Aβ deposition and tau aggregation commence decades prior to overt clinical symptoms, with preferential involvement of brain regions essential for sleep–wake regulation and respiration—namely the brainstem (particularly the locus coeruleus and raphe nuclei), the hypothalamus (orexin/hypocretin neurons), and the basal forebrain (65). Complementing this, experimental animal models have demonstrated that Aβ overexpression elicits sleep fragmentation and perturbed respiratory control (66). In human studies, positron emission tomography (PET) has shown that cortical Aβ burden correlates with diminished slow-wave sleep and increased sleep fragmentation, even among cognitively normal older adults (67). Moreover, tau pathology within the brainstem, which emerges at the earliest stages of Alzheimer's disease (Braak stages I–II), is thought to compromise neuronal populations governing upper airway patency and central respiratory drive, potentially heightening susceptibility to OSA (68). Therefore, the observed association between OSA and neurodegenerative biomarkers may reflect bidirectional relationships: OSA may promote Aβ and tau pathology through hypoxia, sleep fragmentation, and impaired glymphatic clearance; conversely, early AD pathology may disrupt sleep and respiratory regulation, worsening or precipitating OSA (69). The cross-sectional design of most included studies precludes any determination of the temporal sequence of these events. Longitudinal studies with repeated assessments of both OSA status (e.g., polysomnography) and AD biomarkers (e.g., PET, CSF, or plasma) are urgently needed to disentangle the directionality of this relationship.

Beyond these mechanistic considerations, the association between OSA and biomarker alterations may also be modulated by OSA severity and comorbid conditions, which are discussed below.

### 4.4 Association between OSA severity and biomarker alterations

Regarding OSA severity, it is plausible that only moderate-to-severe OSA (AHI ≥15) is associated with significant glymphatic dysfunction and biomarker alterations (70); although most included studies (*n* = 10) pre-dominantly enrolled moderate-to-severe patients and only three included substantial proportions of mild cases, and formal meta-regression was precluded by the fact that only four studies reported severity-stratified data, available data suggest a trend toward larger effect sizes in severe-only studies (e.g., SMD for CSF Aβ42: −1.58 vs. −0.94 in mixed-severity studies), supporting a modulatory role of severity. Furthermore, comorbidities commonly associated with OSA—including hypertension, diabetes, obesity, and cardiovascular disease—are themselves linked to altered Aβ and tau levels (71); however, adjustment for comorbidities across studies was inconsistent (only six studies adjusted for age, sex, and BMI; only two for vascular comorbidities), and residual confounding may contribute to heterogeneity and potentially inflate or mask true associations. Finally, the null findings for *p*-tau and the Aβ42/Aβ40 ratio may also stem from methodological factors: the limited number of studies per outcome (*n* = 3–7) restricts statistical power to detect moderate effect sizes; between-study heterogeneity in OSA severity, assay methods, and population characteristics may obscure true associations detectable in more homogeneous samples; and assay sensitivity for *p*-tau—particularly in blood—varies substantially across platforms, with many studies employing less sensitive ELISA rather than ultra-sensitive SIMOA assays (72). Thus, these null findings should not be interpreted as definitive evidence of no association, but rather as indicating that currently available evidence does not support such an association.

### 4.5 Comparison with prior meta-analyses

Our findings align with previous meta-analyses reporting elevated blood *T*-Aβ and *T*-tau in OSA patients and reduced CSF Aβ40 compared with controls. However, unlike earlier work, the primary analysis of the present study did not show a statistically significant increase in blood Aβ42 (*p* = 0.0506). Nevertheless, a leave-one-out sensitivity analysis indicated that a single study heavily influenced this null finding (44); its exclusion yielded a significant estimate, albeit with substantial residual heterogeneity (*I*^2^ = 79.9%). Therefore, the association between OSA and blood Aβ42 remains inconclusive and requires confirmation in larger, well-standardized cohorts. For blood Aβ40, no significant difference was observed in either the primary or sensitivity analyses (73). Moreover, we identified a significant reduction in CSF Aβ42 among OSA patients, a finding not reported in prior meta-analyses. This discrepancy may be attributable to differences in study populations: previous meta-analyses primarily focused on AD patients, whereas our analysis included a broader spectrum of participants, such as those with spinal cord injury and periodic limb movement disorder (9, 45). This suggests that our results may be more generalizable and highlights the need for future research to examine whether comorbid conditions differentially influence the relationship between OSA and neurodegenerative biomarkers in blood and CSF. Although some included studies stratified biomarker levels by OSA severity, the number was limited, underscoring the importance of future investigations assessing whether biomarker alterations vary according to disease severity.

### 4.6 Limitations, future research directions

This meta-analysis clarifies the association between OSA and neurodegenerative biomarkers in blood and CSF but has several limitations that warrant consideration.

**First**, the vast majority of included studies are cross-sectional in design, inherently limiting causal or temporal inference. Reverse causality remains a possibility—pre-existing neurodegenerative pathology could contribute to sleep-disordered breathing rather than OSA independently driving biomarker changes—and unmeasured confounding cannot be ruled out. Although most included studies conducted biomarker sampling concurrently with OSA diagnosis or within 1 week after diagnosis, effectively reducing the time lag between exposure assessment and outcome measurement, the cross-sectional study design still cannot determine whether biomarker alterations occur prior to or after OSA onset. This ambiguity in temporal sequencing fundamentally constrains causal inference and highlights the need for longitudinal study designs to establish clear temporality between exposures and outcomes. While a small number of existing studies have explored the effects of continuous positive airway pressure (CPAP) intervention on biomarker levels (74), the limited sample size of such studies and their relatively short follow-up periods prevent robust conclusions regarding the reversibility of biomarker alterations.

**Second**, cognitive assessment methods varied across studies, ranging from comprehensive neuropsychological batteries to clinical interviews without standardized tools. While most studies enrolled cognitively normal participants or excluded dementia/MCI, only one study specifically focused on MCI patients. This heterogeneity limits the comparability of findings across studies.

**Third**, substantial heterogeneity remains a key concern for certain biomarkers. According to our GRADE assessments, blood *T*-tau, Aβ40, and Aβ42 exhibited very serious inconsistency (*I*^2^ > 90%), and sensitivity analyses failed to explain its sources fully. Similarly, CSF Aβ42 (*I*^2^ = 77.3%) and CSF *P*-tau (*I*^2^ = 61.3%) also showed serious inconsistency, with prediction intervals crossing or approaching the null. Beyond statistical heterogeneity, considerable clinical heterogeneity exists across studies (75): OSA severity varied substantially (mean AHI ranging from 8.6 to 63.9 events/hour), with different distributions of mild, moderate, and severe cases between cohorts; biomarker assay methods differed markedly (ELISA, SIMOA, or IMR), sample types varied (plasma vs. serum), CSF collection and processing protocols were not uniform, and populations differed in key characteristics including mean age (29.7 to 69.0 years), BMI (24.0 to 35.0 kg/m^2^), comorbidity profiles, and recruitment settings (community-based vs. clinic-based)(76). These clinical heterogeneities likely contributed to the high *I*^2^ values observed and limited the comparability of pooled estimates. While meta-regression revealed that age significantly influenced CSF *T*-tau and *P*-tau levels—consistent with known age-related changes in biomarker metabolism and clearance—the analysis was constrained by the small number of studies per covariate (77), precluding comprehensive adjustment for multiple confounders and reducing statistical power. Consequently, meta-regression results should be viewed as exploratory, and residual confounding from unmeasured or insufficiently powered factors such as obesity, dietary patterns, and sex cannot be excluded (78, 79).

**Fourth**, funnel plot inspection suggested potential publication bias for blood *T*-tau and CSF *P*-tau, warranting cautious interpretation of positive findings for these two biomarkers.

**Fifth**, restricting inclusion to English-language studies may have introduced language bias, and our literature search did not include Scopus; however, given the substantial overlap among the four major databases searched (PubMed, Web of Science, Embase, Cochrane Library) and Scopus, and the small number of included studies (*n* = 13), we consider it unlikely that additional studies from Scopus would change our main conclusions.

**Sixth**, we excluded one study reporting biomarker levels as medians with IQRs because reliable conversion to means and SDs was not possible due to missing minimum/maximum values and unsuccessful author contact. While validated conversion methods exist (14, 15), their accuracy depends on full distributional information (minimum, maximum, and sample size), which was unavailable; this exclusion may have introduced bias, though sensitivity analysis suggested that imputation would not have altered the main conclusions. In addition, the limited number and relatively small sample sizes of the included studies reduce the precision and reliability of our findings, underscoring the need for large-scale, longitudinal cohort studies.

**Finally**, given the pre-dominantly cross-sectional design and the low-to-very-low GRADE certainty for most biomarkers, causal relationships cannot be inferred; the observed associations should be interpreted as correlational rather than causal. The low-to-very-low certainty for most blood biomarkers and the CSF Aβ42/Aβ40 ratio indicates that future studies are likely to alter the magnitude or even direction of these estimates substantially. In contrast, the moderate certainty for CSF Aβ40 and CSF *T*-tau provides more confidence that the direction of effect is unlikely to change, though the magnitude may be refined. These GRADE-based considerations guide our interpretation: while suggestive, the findings should be considered hypothesis-generating rather than conclusive, particularly for biomarkers with very low certainty evidence. Furthermore, the wide prediction intervals observed in several analyses, which crossed the null value, underscore the imprecision of our estimates and further caution against overinterpretation of the pooled effect sizes. Despite these sensitivity analyses, the considerable clinical and methodological heterogeneity across studies, including variations in OSA severity, biomarker assays, and population characteristics, remains an inherent limitation of the current evidence. It should accordingly temper the interpretation of the pooled estimates.

## 5 Conclusion

In summary, the current evidence must be interpreted with considerable caution, as the overall certainty of the findings remains low to very low according to GRADE assessment, and prediction intervals for several key estimates frequently crossed the null value, reflecting substantial uncertainty. Within this exploratory context, this meta-analysis suggests that OSA may be associated with alterations in certain blood and CSF biomarkers, specifically elevated total Aβ and total tau in blood, and reduced Aβ40 and Aβ42 in CSF. However, neither *P*-tau nor the CSF Aβ42/Aβ40 ratio—the biomarkers most specific to AD neuropathology—showed significant changes, indicating that the current evidence does not support a direct association between OSA and established AD-type neurodegeneration. Given these methodological constraints—cross-sectional designs, substantial heterogeneity, modest sample sizes, and low-to-very-low certainty of evidence—these findings should be regarded as strictly hypothesis-generating rather than confirmatory. Future large-scale, prospective longitudinal studies with standardized protocols are urgently needed to determine whether these biomarker alterations represent reversible physiological responses to intermittent hypoxia and sleep fragmentation, or whether they may evolve into genuine neurodegenerative pathology over the long term.

## Data Availability

The original contributions presented in the study are included in the article/supplementary material, further inquiries can be directed to the corresponding authors.
