# Supplementary material for: Associations between obstructive sleep apnea and Alzheimer's disease-related biomarkers (amyloid-β and tau) in blood and cerebrospinal fluid: a systematic review and meta-analysis
Source: Front Neurol. 2026 Sep 21;17:1817426. doi: 10.3389/fneur.2026.1817426 (PMC13634816; doi:10.3389/fneur.2026.1817426)
Supplement: Supplementary file 1 [file Table_1.docx]

**Supplementary file 1: Search strategy**

**Search Strategy for WOS**

| No. | **Search Query** |
| --- | --- |
| **#**1 | Sleep Apnea, Obstructive (Topic) OR Apneas, Obstructive Sleep (Topic) OR Obstructive Sleep Apneas (Topic) OR Sleep Apneas, Obstructive (Topic) OR Apnea, Obstructive Sleep (Topic) OR Sleep Apnea Hypopnea Syndrome (Topic) OR Obstructive Sleep Apnea Syndrome (Topic) OR Obstructive Sleep Apnea (Topic) OR Syndrome, Obstructive Sleep Apnea (Topic) OR Syndrome, Sleep Apnea, Obstructive (Topic) OR Sleep Apnea Syndrome, Obstructive (Topic) OR OSAHS (Topic) OR Upper Airway Resistance Sleep Apnea Syndrome (Topic) OR Syndrome, Upper Airway Resistance, Sleep Apnea (Topic) |
| **#**2 | Amyloid beta Peptides (Topic) OR Amyloid beta-Peptides (Topic) OR beta-Peptides, Amyloid (Topic) OR Alzheimer beta-Protein (Topic) OR Alzheimer beta Protein (Topic) OR beta-Protein, Alzheimer (Topic) OR Alzheimer's ABP (Topic) OR ABP, Alzheimer's (Topic) OR Alzheimer ABP (Topic) OR Alzheimers ABP (Topic) OR Alzheimer's Amyloid Fibril Protein (Topic) OR Amyloid AD-AP (Topic) OR AD-AP, Amyloid (Topic) OR Amyloid AD AP (Topic) OR Amyloid beta-Peptide (Topic) OR Amyloid beta Peptide (Topic) OR beta-Peptide, Amyloid (Topic) OR Amyloid beta-Protein (Topic) OR Amyloid beta Protein (Topic) OR beta-Protein, Amyloid (Topic) OR Amyloid beta-Proteins (Topic) OR Amyloid beta Proteins (Topic) OR beta-Proteins, Amyloid (Topic) OR Amyloid Fibril Protein, Alzheimer's (Topic) OR Amyloid Protein A4 (Topic) OR Protein A4, Amyloid (Topic) OR beta Amyloid (Topic) OR Amyloid, beta (Topic) OR beta-Amyloid Protein (Topic) OR beta Amyloid Protein (Topic) OR Protein, beta-Amyloid (Topic) |
| **#**3 | tau Proteins (Topic) OR Proteins, tau (Topic) OR tau Protein (Topic) OR Protein, tau (Topic) |
| **#**4 | #2 OR #3 |
| **#**5 | #4 AND #1 |

**Search Strategy for Embase**

| **No.** | **Query** |
| --- | --- |
| #11 | #3 AND #10 |
| #10 | #6 OR #9 |
| #9 | #7 OR #8 |
| #8 | 'microtubule associated protein tau':ab,ti OR 'microtubule associated tau protein':ab,ti OR 'microtubule protein tau':ab,ti OR 'tau factor':ab,ti OR 'tau proteins':ab,ti OR 'tau protein':ab,ti |
| #7 | 'tau protein'/exp |
| #6 | #4 OR #5 |
| #5 | 'a beta peptide':ab,ti OR 'a beta protein':ab,ti OR 'abeta peptide':ab,ti OR 'abeta protein':ab,ti OR 'amyloid beta peptide':ab,ti OR 'amyloid beta peptides':ab,ti OR 'amyloid beta-peptides':ab,ti OR 'amyloid beta-protein':ab,ti OR 'amyloid peptide beta':ab,ti OR 'amyloid protein beta':ab,ti OR 'beta amyloid peptide':ab,ti OR 'beta amyloid protein':ab,ti OR 'amyloid beta protein':ab,ti |
| #4 | 'amyloid beta protein'/exp |
| #3 | #1 OR #2 |
| #2 | 'obstructive apnea':ab,ti OR 'obstructive apnea during sleep':ab,ti OR 'obstructive apneas during sleep':ab,ti OR 'obstructive apnoea':ab,ti OR 'obstructive sleep apnea hypopnea syndrome':ab,ti OR ('obstructive sleep apnea hypopnea syndrome':ab,ti AND osahs:ab,ti) OR 'obstructive sleep apnea syndrome':ab,ti OR 'obstructive sleep apnea-hypopnea':ab,ti OR 'obstructive sleep apneas':ab,ti OR 'obstructive sleep apnoe':ab,ti OR 'obstructive sleep apnoe-hypopnoe syndrome':ab,ti OR 'obstructive sleep apnoea':ab,ti OR 'obstructive sleep apnoea hypopnoea syndrome':ab,ti OR 'obstructive sleep apnoea syndrome':ab,ti OR 'obstructive sleep-disordered breathing':ab,ti OR (osahs:ab,ti AND 'sleep apnea':ab,ti) OR 'sleep apnea, obstructive':ab,ti OR 'sleep apnoea, obstructive':ab,ti OR 'obstructive sleep apnea':ab,ti |
| #1 | 'obstructive sleep apnea'/exp |

**Search Strategy for Cochrane Library**

| **No.** | **Query** |
| --- | --- |
| #1 | MeSH descriptor: [Sleep Apnea, Obstructive] explode all trees |
| #2 | (Sleep Apnea, Obstructive):ti,ab,kw OR (Sleep Apnea Hypopnea Syndrome):ti,ab,kw OR (OSAHS):ti,ab,kw OR (Sleep Apneas, Obstructive):ti,ab,kw OR (Syndrome, Obstructive Sleep Apnea):ti,ab,kw |
| #3 | (Apnea, Obstructive Sleep):ti,ab,kw OR (Apneas, Obstructive Sleep):ti,ab,kw OR (Obstructive Sleep Apnea Syndrome):ti,ab,kw OR (Sleep Apnea Syndrome, Obstructive):ti,ab,kw OR (Syndrome, Sleep Apnea, Obstructive):ti,ab,kw |
| #4 | (Obstructive Sleep Apneas):ti,ab,kw OR (Obstructive Sleep Apnea):ti,ab,kw OR (Upper Airway Resistance Sleep Apnea Syndrome):ti,ab,kw OR (Syndrome, Upper Airway Resistance, Sleep Apnea):ti,ab,kw |
| #5 | #1 OR #2 OR #3 OR #4 |
| #6 | MeSH descriptor: [Amyloid beta-Peptides] explode all trees |
| #7 | (Amyloid beta-Peptides):ti,ab,kw OR (Amyloid beta Peptide):ti,ab,kw OR (beta Amyloid):ti,ab,kw OR (Amyloid AD AP):ti,ab,kw OR (Amyloid beta Protein):ti,ab,kw |
| #8 | (Alzheimer's ABP):ti,ab,kw OR (Alzheimer's Amyloid Fibril Protein):ti,ab,kw OR (Protein A4, Amyloid):ti,ab,kw OR (beta-Protein, Amyloid):ti,ab,kw OR (Amyloid, beta):ti,ab,kw |
| #9 | (Alzheimer beta Protein):ti,ab,kw OR (Amyloid AD-AP):ti,ab,kw OR (Amyloid beta-Peptide):ti,ab,kw OR (AD-AP, Amyloid):ti,ab,kw OR (beta Amyloid Protein):ti,ab,kw |
| #10 | (Protein, beta-Amyloid):ti,ab,kw OR (Amyloid beta Peptides):ti,ab,kw OR (Alzheimer beta-Protein):ti,ab,kw OR (Amyloid beta Proteins):ti,ab,kw OR (Alzheimer ABP):ti,ab,kw |
| #11 | (Alzheimers ABP):ti,ab,kw OR (ABP, Alzheimer's):ti,ab,kw OR (Amyloid Fibril Protein, Alzheimer's):ti,ab,kw OR (beta-Amyloid Protein):ti,ab,kw OR (Amyloid Protein A4):ti,ab,kw |
| #12 | (beta-Proteins, Amyloid):ti,ab,kw OR (Amyloid beta-Protein):ti,ab,kw OR (beta-Protein, Alzheimer):ti,ab,kw OR (beta-Peptides, Amyloid):ti,ab,kw OR (Amyloid beta-Proteins):ti,ab,kw |
| #13 | (beta-Peptide, Amyloid):ti,ab,kw |
| #14 | #6 OR #7 OR #8 OR #9 OR #10 OR #11 OR #12 OR #13 |
| #15 | MeSH descriptor: [tau Proteins] explode all trees |
| #16 | (Proteins, tau):ti,ab,kw OR (tau Proteins):ti,ab,kw OR (tau Protein):ti,ab,kw OR (Protein, tau):ti,ab,kw |
| #17 | #15 OR #16 |
| #18 | #14 OR #17 |
| #19 | #5 AND #18 |

**Search Strategy for Pubmed**

| **No.** | **Query** |
| --- | --- |
| #11 | ((((((tau Proteins[Title/Abstract]) OR (Proteins, tau[Title/Abstract])) OR (tau Protein[Title/Abstract])) OR (Protein, tau[Title/Abstract])) OR ("tau Proteins"[Mesh])) OR (("Amyloid beta-Peptides"[Mesh]) OR (((((((((((((((((((((((((((((((Amyloid beta-Peptides[Title/Abstract]) OR (Amyloid beta Peptides[Title/Abstract])) OR (beta-Peptides, Amyloid[Title/Abstract])) OR (Alzheimer beta-Protein[Title/Abstract])) OR (Alzheimer beta Protein[Title/Abstract])) OR (beta-Protein, Alzheimer[Title/Abstract])) OR (Alzheimer's ABP[Title/Abstract])) OR (ABP, Alzheimer's[Title/Abstract])) OR (Alzheimer ABP[Title/Abstract])) OR (Alzheimers ABP[Title/Abstract])) OR (Alzheimer's Amyloid Fibril Protein[Title/Abstract])) OR (Amyloid AD-AP[Title/Abstract])) OR (AD-AP, Amyloid[Title/Abstract])) OR (Amyloid AD AP[Title/Abstract])) OR (Amyloid beta-Peptide[Title/Abstract])) OR (Amyloid beta Peptide[Title/Abstract])) OR (beta-Peptide, Amyloid[Title/Abstract])) OR (Amyloid beta-Protein[Title/Abstract])) OR (Amyloid beta Protein[Title/Abstract])) OR (beta-Protein, Amyloid[Title/Abstract])) OR (Amyloid beta-Proteins[Title/Abstract])) OR (Amyloid beta Proteins[Title/Abstract])) OR (beta-Proteins, Amyloid[Title/Abstract])) OR (Amyloid Fibril Protein, Alzheimer's[Title/Abstract])) OR (Amyloid Protein A4[Title/Abstract])) OR (Protein A4, Amyloid[Title/Abstract])) OR (beta Amyloid[Title/Abstract])) OR (Amyloid, beta[Title/Abstract])) OR (beta-Amyloid Protein[Title/Abstract])) OR (beta Amyloid Protein[Title/Abstract])) OR (Protein, beta-Amyloid[Title/Abstract])))) AND ((((((((((((((Sleep Apnea, Obstructive[Title/Abstract]) OR (Apneas, Obstructive Sleep[Title/Abstract])) OR (Obstructive Sleep Apneas[Title/Abstract])) OR (Sleep Apneas, Obstructive[Title/Abstract])) OR (Apnea, Obstructive Sleep[Title/Abstract])) OR (Sleep Apnea Hypopnea Syndrome[Title/Abstract])) OR (Obstructive Sleep Apnea Syndrome[Title/Abstract])) OR (Obstructive Sleep Apnea[Title/Abstract])) OR (Syndrome, Obstructive Sleep Apnea[Title/Abstract])) OR (Syndrome, Sleep Apnea, Obstructive[Title/Abstract])) OR (Sleep Apnea Syndrome, Obstructive[Title/Abstract])) OR (OSAHS[Title/Abstract])) OR (Upper Airway Resistance Sleep Apnea Syndrome[Title/Abstract])) OR (Syndrome, Upper Airway Resistance, Sleep Apnea[Title/Abstract])) |
| #10 | (((((tau Proteins[Title/Abstract]) OR (Proteins, tau[Title/Abstract])) OR (tau Protein[Title/Abstract])) OR (Protein, tau[Title/Abstract])) OR ("tau Proteins"[Mesh])) OR (("Amyloid beta-Peptides"[Mesh]) OR (((((((((((((((((((((((((((((((Amyloid beta-Peptides[Title/Abstract]) OR (Amyloid beta Peptides[Title/Abstract])) OR (beta-Peptides, Amyloid[Title/Abstract])) OR (Alzheimer beta-Protein[Title/Abstract])) OR (Alzheimer beta Protein[Title/Abstract])) OR (beta-Protein, Alzheimer[Title/Abstract])) OR (Alzheimer's ABP[Title/Abstract])) OR (ABP, Alzheimer's[Title/Abstract])) OR (Alzheimer ABP[Title/Abstract])) OR (Alzheimers ABP[Title/Abstract])) OR (Alzheimer's Amyloid Fibril Protein[Title/Abstract])) OR (Amyloid AD-AP[Title/Abstract])) OR (AD-AP, Amyloid[Title/Abstract])) OR (Amyloid AD AP[Title/Abstract])) OR (Amyloid beta-Peptide[Title/Abstract])) OR (Amyloid beta Peptide[Title/Abstract])) OR (beta-Peptide, Amyloid[Title/Abstract])) OR (Amyloid beta-Protein[Title/Abstract])) OR (Amyloid beta Protein[Title/Abstract])) OR (beta-Protein, Amyloid[Title/Abstract])) OR (Amyloid beta-Proteins[Title/Abstract])) OR (Amyloid beta Proteins[Title/Abstract])) OR (beta-Proteins, Amyloid[Title/Abstract])) OR (Amyloid Fibril Protein, Alzheimer's[Title/Abstract])) OR (Amyloid Protein A4[Title/Abstract])) OR (Protein A4, Amyloid[Title/Abstract])) OR (beta Amyloid[Title/Abstract])) OR (Amyloid, beta[Title/Abstract])) OR (beta-Amyloid Protein[Title/Abstract])) OR (beta Amyloid Protein[Title/Abstract])) OR (Protein, beta-Amyloid[Title/Abstract]))) |
| #9 | ((((tau Proteins[Title/Abstract]) OR (Proteins, tau[Title/Abstract])) OR (tau Protein[Title/Abstract])) OR (Protein, tau[Title/Abstract])) OR ("tau Proteins"[Mesh]) |
| #8 | (((tau Proteins[Title/Abstract]) OR (Proteins, tau[Title/Abstract])) OR (tau Protein[Title/Abstract])) OR (Protein, tau[Title/Abstract]) |
| #7 | "tau Proteins"[Mesh] |
| #6 | ("Amyloid beta-Peptides"[Mesh]) OR (((((((((((((((((((((((((((((((Amyloid beta-Peptides[Title/Abstract]) OR (Amyloid beta Peptides[Title/Abstract])) OR (beta-Peptides, Amyloid[Title/Abstract])) OR (Alzheimer beta-Protein[Title/Abstract])) OR (Alzheimer beta Protein[Title/Abstract])) OR (beta-Protein, Alzheimer[Title/Abstract])) OR (Alzheimer's ABP[Title/Abstract])) OR (ABP, Alzheimer's[Title/Abstract])) OR (Alzheimer ABP[Title/Abstract])) OR (Alzheimers ABP[Title/Abstract])) OR (Alzheimer's Amyloid Fibril Protein[Title/Abstract])) OR (Amyloid AD-AP[Title/Abstract])) OR (AD-AP, Amyloid[Title/Abstract])) OR (Amyloid AD AP[Title/Abstract])) OR (Amyloid beta-Peptide[Title/Abstract])) OR (Amyloid beta Peptide[Title/Abstract])) OR (beta-Peptide, Amyloid[Title/Abstract])) OR (Amyloid beta-Protein[Title/Abstract])) OR (Amyloid beta Protein[Title/Abstract])) OR (beta-Protein, Amyloid[Title/Abstract])) OR (Amyloid beta-Proteins[Title/Abstract])) OR (Amyloid beta Proteins[Title/Abstract])) OR (beta-Proteins, Amyloid[Title/Abstract])) OR (Amyloid Fibril Protein, Alzheimer's[Title/Abstract])) OR (Amyloid Protein A4[Title/Abstract])) OR (Protein A4, Amyloid[Title/Abstract])) OR (beta Amyloid[Title/Abstract])) OR (Amyloid, beta[Title/Abstract])) OR (beta-Amyloid Protein[Title/Abstract])) OR (beta Amyloid Protein[Title/Abstract])) OR (Protein, beta-Amyloid[Title/Abstract])) |
| #5 | ((((((((((((((((((((((((((((((Amyloid beta-Peptides[Title/Abstract]) OR (Amyloid beta Peptides[Title/Abstract])) OR (beta-Peptides, Amyloid[Title/Abstract])) OR (Alzheimer beta-Protein[Title/Abstract])) OR (Alzheimer beta Protein[Title/Abstract])) OR (beta-Protein, Alzheimer[Title/Abstract])) OR (Alzheimer's ABP[Title/Abstract])) OR (ABP, Alzheimer's[Title/Abstract])) OR (Alzheimer ABP[Title/Abstract])) OR (Alzheimers ABP[Title/Abstract])) OR (Alzheimer's Amyloid Fibril Protein[Title/Abstract])) OR (Amyloid AD-AP[Title/Abstract])) OR (AD-AP, Amyloid[Title/Abstract])) OR (Amyloid AD AP[Title/Abstract])) OR (Amyloid beta-Peptide[Title/Abstract])) OR (Amyloid beta Peptide[Title/Abstract])) OR (beta-Peptide, Amyloid[Title/Abstract])) OR (Amyloid beta-Protein[Title/Abstract])) OR (Amyloid beta Protein[Title/Abstract])) OR (beta-Protein, Amyloid[Title/Abstract])) OR (Amyloid beta-Proteins[Title/Abstract])) OR (Amyloid beta Proteins[Title/Abstract])) OR (beta-Proteins, Amyloid[Title/Abstract])) OR (Amyloid Fibril Protein, Alzheimer's[Title/Abstract])) OR (Amyloid Protein A4[Title/Abstract])) OR (Protein A4, Amyloid[Title/Abstract])) OR (beta Amyloid[Title/Abstract])) OR (Amyloid, beta[Title/Abstract])) OR (beta-Amyloid Protein[Title/Abstract])) OR (beta Amyloid Protein[Title/Abstract])) OR (Protein, beta-Amyloid[Title/Abstract]) |
| #4 | "Amyloid beta-Peptides"[Mesh] |
| #3 | ("Sleep Apnea, Obstructive"[Mesh]) OR ((((((((((((((Sleep Apnea, Obstructive[Title/Abstract]) OR (Apneas, Obstructive Sleep[Title/Abstract])) OR (Obstructive Sleep Apneas[Title/Abstract])) OR (Sleep Apneas, Obstructive[Title/Abstract])) OR (Apnea, Obstructive Sleep[Title/Abstract])) OR (Sleep Apnea Hypopnea Syndrome[Title/Abstract])) OR (Obstructive Sleep Apnea Syndrome[Title/Abstract])) OR (Obstructive Sleep Apnea[Title/Abstract])) OR (Syndrome, Obstructive Sleep Apnea[Title/Abstract])) OR (Syndrome, Sleep Apnea, Obstructive[Title/Abstract])) OR (Sleep Apnea Syndrome, Obstructive[Title/Abstract])) OR (OSAHS[Title/Abstract])) OR (Upper Airway Resistance Sleep Apnea Syndrome[Title/Abstract])) OR (Syndrome, Upper Airway Resistance, Sleep Apnea[Title/Abstract])) |
| #2 | (((((((((((((Sleep Apnea, Obstructive[Title/Abstract]) OR (Apneas, Obstructive Sleep[Title/Abstract])) OR (Obstructive Sleep Apneas[Title/Abstract])) OR (Sleep Apneas, Obstructive[Title/Abstract])) OR (Apnea, Obstructive Sleep[Title/Abstract])) OR (Sleep Apnea Hypopnea Syndrome[Title/Abstract])) OR (Obstructive Sleep Apnea Syndrome[Title/Abstract])) OR (Obstructive Sleep Apnea[Title/Abstract])) OR (Syndrome, Obstructive Sleep Apnea[Title/Abstract])) OR (Syndrome, Sleep Apnea, Obstructive[Title/Abstract])) OR (Sleep Apnea Syndrome, Obstructive[Title/Abstract])) OR (OSAHS[Title/Abstract])) OR (Upper Airway Resistance Sleep Apnea Syndrome[Title/Abstract])) OR (Syndrome, Upper Airway Resistance, Sleep Apnea[Title/Abstract]) |
| #1 | "Sleep Apnea, Obstructive"[Mesh] |
